# An electron-hole rich dual-site nickel catalyst for efficient photocatalytic overall water splitting

**DOI:** 10.1038/s41467-023-37358-3

**Published:** 2023-03-29

**Authors:** Xiaoqing Yan, Mengyang Xia, Hanxuan Liu, Bin Zhang, Chunran Chang, Lianzhou Wang, Guidong Yang

**Affiliations:** 1 School of Chemical Engineering and Technology, Xi’an Jiaotong University, 710049 Xi’an, P.R. China; 2 College of Physics and Optoelectronic Engineering, Shenzhen University, 518060 Shenzhen, P.R. China; 3 School of Chemical Engineering, and Australian Institute for Bioengineering and Nanotechnology, The University of Queensland, Brisbane, QLD 4072 Australia

**Keywords:** Photocatalysis, Photocatalysis, Photocatalysis, Hydrogen fuel

## Abstract

Photocatalysis offers an attractive strategy to upgrade H_2_O to renewable fuel H_2_. However, current photocatalytic hydrogen production technology often relies on additional sacrificial agents and noble metal cocatalysts, and there are limited photocatalysts possessing overall water splitting performance on their own. Here, we successfully construct an efficient catalytic system to realize overall water splitting, where hole-rich nickel phosphides (Ni_2_P) with polymeric carbon-oxygen semiconductor (PCOS) is the site for oxygen generation and electron-rich Ni_2_P with nickel sulfide (NiS) serves as the other site for producing H_2_. The electron-hole rich Ni_2_P based photocatalyst exhibits fast kinetics and a low thermodynamic energy barrier for overall water splitting with stoichiometric 2:1 hydrogen to oxygen ratio (150.7 μmol h^−1^ H_2_ and 70.2 μmol h^−1^ O_2_ produced per 100 mg photocatalyst) in a neutral solution. Density functional theory calculations show that the co-loading in Ni_2_P and its hybridization with PCOS or NiS can effectively regulate the electronic structures of the surface active sites, alter the reaction pathway, reduce the reaction energy barrier, boost the overall water splitting activity. In comparison with reported literatures, such photocatalyst represents the excellent performance among all reported transition-metal oxides and/or transition-metal sulfides and is even superior to noble metal catalyst.

## Abstract (web summary)

Vast majority of photocatalysts for hydrogen production relies on additional sacrificial agents and noble metal cocatalysts. It is of great importance yet challenging to achieve photocatalytic overall water splitting with decent performance. Here, the authors report Ni_2_P based photocatalyst assisted by H_2_O_2_-craking reaction for overall water splitting with H_2_ and O_2_ production of 1507 μmol h^−1^ g^−1^ H_2_ and 702 μmol h^−1^ g^−1^.

## Introduction

Solar-driven overall water splitting by photocatalysis is a promising technology, capable of achieving efficient and clean solar energy conversion to hydrogen fuel^1–3^. However, current photocatalytic hydrogen production technology rarely demonstrates both H_2_ and O_2_ production performance, with the vast majority of recent photocatalysis research being focused on the more facile half-reactions using sacrificial agents to scavenge photogenerated holes or electrons in the photocatalysts. The main challenges in achieving overall water splitting include how to kinetically facilitate a four-electron process (2H_2_O → 2H_2_ + O_2_) and how to overcome a high (1.8 eV) thermodynamic energy barrier (including overpotential), with the rate-determining step being the more difficult oxidation half-reaction^4–8^. The rational design of novel photocatalysts to alter the kinetic pathway and reduce the thermodynamic energy barrier in H_2_O oxidation is especially important to achieve overall water splitting. Recently, water oxidation via a two-step/two-electron process (2H_2_O + 2e^–^/h^+^→H_2_ + H_2_O_2_; H_2_O_2_ → H_2_O + 0.5O_2_), where water undergoes a redox reaction to form hydrogen and hydrogen peroxide, the latter of which subsequently decomposes to form water and oxygen, has been explored as an alternative strategy for overall water splitting from a kinetics standpoint, and has shown exciting progress^9–12^. For example, Wu et al.^12^ have achieved a two-electron/two-step overall water splitting pathway on transition-metal-doped structure catalysts, despite that the activity is not satisfactory. In addition, research indicates that creating surface redox-active sites offers an efficient and simple way to lower the thermodynamic energy barrier of the H_2_O oxidation process^13^. Using electron-rich carbon materials as a dopant is a simple selection^14,15^. For example, Qiao et al.^8^ reported a nitrogen-doped carbon photocatalyst using a chemical vapor deposition technique, which can vastly reduce the overpotential for the oxygen evolution reaction. However, to date, there are relatively few studies on enhancing the activity by altering both the kinetics and thermodynamics of the catalysts.

Modulating electronic structures of the active sites on metal-based catalysts has been considered as an effective approach to enhance the catalytic activity^16^. In industrial heterogeneous catalysis, the electronic structure can be effectively adjusted by the metal–support interactions, which can result in realigning the molecular energy levels and modulating d-band structure of metal-based catalysts. The d-orbital electrons can change the adsorption energy and reduce the activation energy barrier^17–19^. More recently, nickel phosphide (Ni_2_P) has attracted growing attention due to its fascinating electronic properties, as it can change the kinetic process of water splitting^5,15,20^, and its tunable bandgap can also lead to Ni_2_P with sufficient redox potential to overcome the thermodynamic barrier^21–25^. These advantages make Ni_2_P a good candidate to achieve overall photocatalytic water splitting^26,27^. However, when Ni_2_P is used as the main catalyst, it suffers from poor photocatalytic activity as a result of limited availability of active sites and poor stability against oxygen^28,29^. In this work, we present an electron-hole rich dual-site nickel-based catalytic systems, consisting of Ni_2_P with a nickel sulfide site and polymeric carbon–oxygen semiconductor site (Ni_2_P/NiS@PCOS). In the system of Ni_2_P/NiS@PCOS, the use of NiS greatly increases photogenerated charge carrier separation efficiencies and hydrogen production activity^30^. And importantly, the PCOS coated on the Ni_2_P is found to not only reduce the overpotential, owing to the enhanced charge density resulting from the N and O doping^3,9,31^, but also lead to electron migration at the PCOS/Ni_2_P interface due to its strong electron donating ability, which would create more active sites for the H_2_O oxidation process^32^. Benefitting from the synergistic effects of NiS and PCOS, the Ni_2_P/NiS@PCOS photocatalyst readily dissociates H_2_O to *OH (* + H_2_O → *OH + H^+^ + e^–^), which then produces H_2_O_2_ through the well-known two-electron pathway, owing to a low energy barrier (1.22 eV for H_2_O_2_ production vs. 2.35 eV for O_2_ production), next H_2_O_2_ could be easily broken down to release oxygen. As result of making the sample exhibit an overall water splitting performance with nearly stoichiometric oxygen (70.2 μmol h^−1^), and hydrogen evolution (150.7 μmol h^−1^) in the photoreaction. It is the best activity among all reported transition-metal oxides and/or transition-metal sulfides under the same conditions. The finding will pave the way for the development of efficient semiconductor photocatalysts that don’t require sacrificial electron donors or expensive metal cocatalysts to achieve efficient overall water splitting.

## Results

### Characterizations of as-prepared photocatalysts

**Fig. 1:**
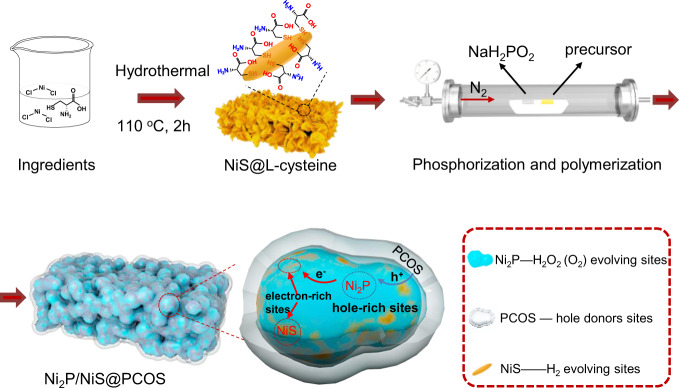
Schematic diagram illustrating the synthetic route of Ni_2_P/NiS@PCOS. The gray, bule, and orange represent polymeric carbon–oxygen semiconductor (PCOS), nickel phosphides (Ni_2_P), and nickel sulfide (NiS), respectively.

**Fig. 2:**
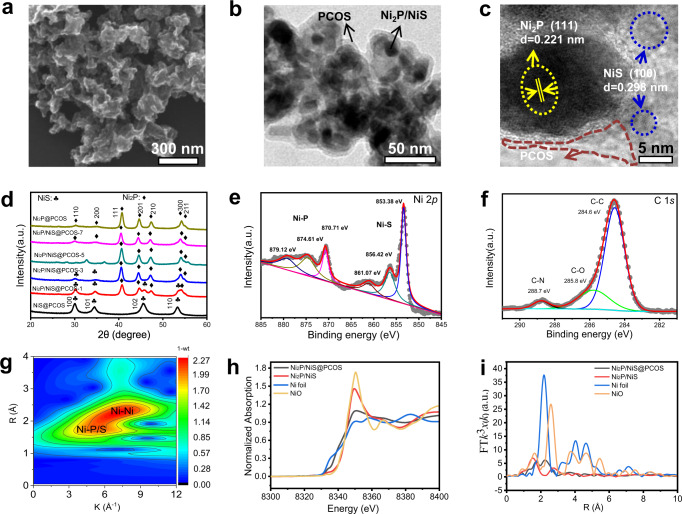
Morphology and structural characterizations of typical samples. **a** SEM images of Ni_2_P/NiS@PCOS; **b** TEM images of Ni_2_P/NiS@PCOS; **c** high-resolution TEM (HRTEM) images of Ni_2_P/NiS@PCOS; **d** XRD patterns of NiS@PCOS and Ni_2_P/NiS@PCOS composites; **e**, **f** XPS of Ni_2_P/NiS@PCOS; **g** Wavelet transform of Ni–K-edge EXAFS in Ni_2_P/NiS@PCOS; **h** XANES spectra of Ni, NiO standards, Ni_2_P/NiS and Ni_2_P/NiS@PCOS. **i** EXAFS spectra of Ni, NiO standards, Ni_2_P/NiS, and Ni_2_P/NiS@PCOS.

As shown in Fig. 1 and Supplementary Fig. 1, in situ adsorption of l-cysteine on the surface of the NiS nanosheets was realized via solid metal-sulfydryl bond interactions in the hydrothermal process, where the Ni^2+^ ions were linked by l-cysteine, which acted as a bifunctional surface modifier^33,34^. Under high temperature, the PH_3_ released from NaH_2_PO_2_ reduced the NiS into Ni_2_P. And the in situ adsorption of l-cysteine on surface of NiS through complex polymerization results in a doped polymeric carbon–oxygen semiconductor, which will be discussed later. In addition, we tailored the microstructures of Ni_2_P by changing the amount of NaH_2_PO_2_ powder. From the XRD data (Fig. 2d), the change of the photocatalyst from NiS@l-cysteine to Ni_2_P/NiS@PCOS and Ni_2_P@PCOS after the phosphorization at 350 °C for 3 h was clearly observed. The intensity of the XRD peaks of Ni_2_P was gradually enhanced during the solid-state reaction and reconstructed collapse of nanosheets at high reaction temperature. A close inspection of XRD pattern shows that the position of (111) peak was shifted slightly to a lower 2*θ* value, indicating an increase of structural defects of the photocatalyst during the phosphating process^35^. In the system of Ni_2_P/NiS@PCOS heterojunction, no obvious diffraction peaks of polymeric carbon–oxygen semiconductor were observed due to its low concentration.

SEM (Supplementary Fig. 2a, b) and TEM images (Supplementary Fig. 3a–d) of NiS@l-cysteine showed a thin nanosheet morphology with an average width of ≈500 nm. After the phosphorization process, nanoparticles with a diameter of around 30 nm were gradually and disorderly loaded on the side of the NiS nanosheets, while the NiS nanosheets concurrently became nanoparticles, as shown in SEM (Fig. 2a) and TEM images (Fig. 2b and Supplementary Fig. 4a–d). The TEM image further confirms that there is a thin layer of polymeric carbon–oxygen semiconductor coating on the surface of Ni_2_P/NiS. In the HRTEM image (Fig. 2c), the lattice spacing (d values) of the nanoparticle was tested to be 0.221 nm, assigned to the (111) plane of Ni_2_P, with an obvious interface contact with NiS (0.298 nm, 100 plane) observed, indicating that the Ni_2_P nanoparticles grew on the side of the NiS. This result highlighted several nano-size heterojunctions between Ni_2_P and NiS, which could accelerate the photogenerated charge carrier migration and separation. The average thickness of the PCOS, analyzed using TEM (Fig. 2b and Supplementary Fig. 4c) images, was 1–5 nm. And the average pore size of Ni_2_P/NiS with PCOS is concentrated around 1.33 nm by the BET testing (Supplementary Fig. 5 and Supplementary Table 1), which can ensure the water molecules migration. From SEM/TEM (Supplementary Fig. 6a–d), HRTEM (Fig. 2c) and STEM elemental mapping spectra of Ni_2_P/NiS@PCOS (Supplementary Fig. 7), it was shown that the PCOS is a high crystalline polymeric carbon–oxygen material with N and S. Through the above analysis, the detailed structure of the photocatalyst can be confirmed as the component of Ni_2_P with NiS and PCOS.

To further understand the role of PCOS in the system of Ni_2_P/NiS@PCOS heterojunction, the structure of pure PCOS, which was prepared by calcining the pure l-cysteine under the same conditions, was analyzed. It was found that the PCOS exhibited two typical Raman bands at 1359 cm^−1^ and 1571 cm^−1^, which represent the defect structure (D band) and the degree of graphitization (G band) (Supplementary Fig. 8). The average *I*_D_:*I*_G_ value was determined to be about 0.80, indicating this material possessed abundant defect sites and a disordered structure^36^. It is inferred that the nitrogen and sulfur atom were successfully doped into the PCOS. A similar result was also confirmed by Solid-state ^13^C-nuclear magnetic resonance (NMR) measurements (Supplementary Fig. 9). From the XRD analysis (Supplementary Fig. 10), PCOS shows good crystallinity, with the classic peaks of amorphous carbon being clearly identified. The FT-IR spectrum (Supplementary Fig. 11) demonstrated the presence of C–N, C = C, C–S, and C–O groups in the PCOS^37^.

To further examine the chemical components and electronic states of elements in the Ni_2_P/NiS@PCOS heterojunction, X-ray photoelectron spectroscopy (XPS) measurements were performed, shown in Fig. 2e, f and Supplementary Fig. 12a, b. The Ni 2*p* XPS spectrum (Fig. 2e) showed two main peaks associated with NiS, located at the binding energies of 853.38 eV and 856.42 eV. Two additional peaks could be observed at higher binding energies of 870.71 eV and 874.61 eV, which were assigned to Ni_2_P. The results indicate clearly that the P has partially replaced S in the surface of NiS to form the Ni_2_P. Two other peaks observed at 879.12 eV and 861.07 eV are shake-up satellites^38^. Fig. 2f showed the C XPS data, the peak of C 1 *s* at the binding energy of 284.6 eV, 285.8 eV, and 288.7 eV were assigned to the C–C, C–O, and C–N, respectively, inferring that the polymeric carbon–oxygen semiconductor contains abundant nitrogen and oxygen surface functional groups. Further insight into the formation of PCOS was obtained by calcining l-cysteine at different temperatures and analyzing the resultant XPS (C 1 *s*, N 2*p* and S 2*p*) data, shown in Supplementary Fig. 13. The results indicate that the polymeric carbon–oxygen semiconductor was comprised of 8.14% N and 2.58% S, which was a higher N,S-doping efficiency for our synthetic method than most previously reported methods^33^. In addition, the high-resolution spectrum of N can be divided into two peaks located at 398.0 eV and 400.8 eV after peak devolution, which can be correspondingly indexed to pyridinic N and graphitic N, with an estimated content of 5.24% and 2.90%, respectively. The high-resolution S 2*p* peaks can be resolved into two peaks associated with C–S–C (163.2 eV for 2 *p*_3/2_,164.4 eV for S 2*p*_1/2_) species^36^. According to previous reports^36,39,40^, the high content of N or S-doping can enhance the charge density of materials, subsequently improving the photocatalytic activity. The detailed atomic percentages are summarized in Supplementary Tables 2–4.

X-ray absorption spectroscopy (XAS) analysis was performed for further checking the electronic structures and directly investigating the coordinating environment of Ni species in Ni_2_P/NiS with and without PCOS (Fig. 2g–i, Supplementary Fig. 14, and Supplementary Table 5). Notably in the X-ray absorption near-edge structure (XANES) spectrum (Fig. 2h), the red-line intensity of Ni at 8350 eV in Ni_2_P/NiS is higher than that of Ni_2_P/NiS@PCOS, and closer to that of NiO standards. Estimation of the Ni valence for both samples are +0.4 and +1.3, respectively. The positively charged Ni suggests electronic interaction between Ni and other elements, and different valent values indicate varied coordination. Extended X-ray absorption fine structure spectroscopy (EXAFS) in Fig. 2i provided key evidence for the local environment of Ni species. It is found that in the Wavelet transform spectra (Supplementary Fig. 14a–c), almost no Ni–P/S contribution at ~2.23 Å was observed in the *k*^2^-weighted EXAFS at Ni K–edge of Ni_2_P/NiS, and the Ni–O contribution at around 2.23 Å dominated. While with the introduction of PCOS, the EXAFS spectrum displayed obvious Ni-N/S contribution but no Ni–O or Ni–C bonding (Fig. 2g), strongly indicating that Ni existed predominantly as Ni–P and Ni–S coordinating sites. Detailed EXAFS fitting results are provided in Supplementary Table 5. Without the protection of PCOS, the average Ni–O coordination number reached 5.1 which is close to 6 in Ni–O, confirming that the as-prepared Ni_2_P/NiS are easily oxidized into NiO, which may lose its catalytic performance. In Ni_2_P/NiS@PCOS, an average coordination number of 4.7 for Ni–S/P bonding and 2.9 for Ni–Ni bonding have been obtained, proving that Ni in this sample exists mainly in the form of Ni_2_P/NiS and that PCOS plays a significant role in preventing Ni_2_P/NiS from oxidation ( +0.4 vs. +1.3), but no Ni–C/N/O contribution manifests. It was inferred that the electron transfer between PCOS and Ni_2_P/NiS can directly interact via Ni–S–C, therefore the Ni atom showed excellent electron-rich properties. In the system of Ni_2_P/NiS@PCOS, PCOS not only serves as semiconductor during the photocatalytic process, but also plays a significant role in preserving the electronic structure and reactivity of Ni_2_P/NiS with good stability.

### Photocatalytic overall water splitting

**Fig. 3:**
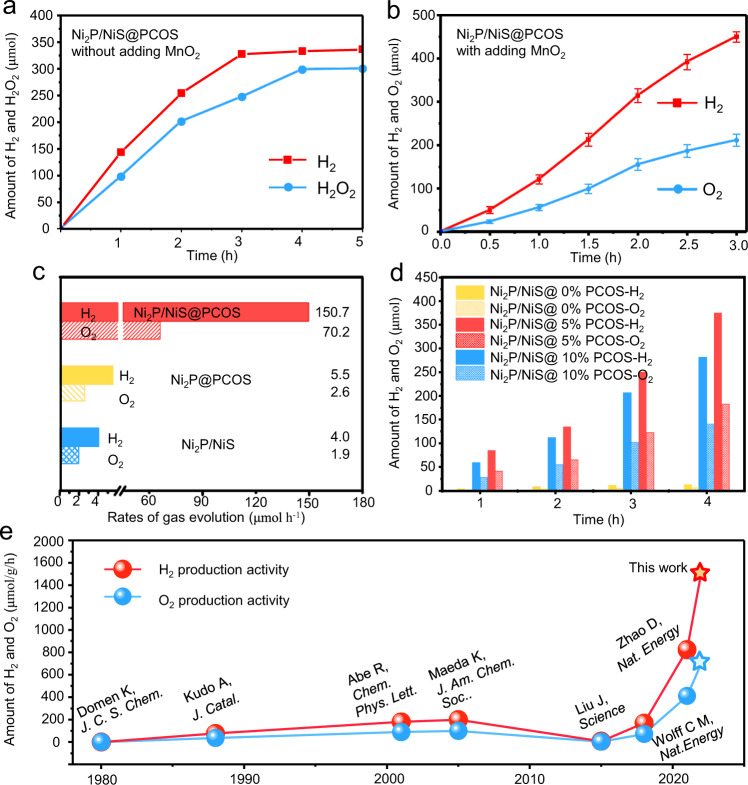
Photocatalytic performance of overall water splitting. Time-dependent photocatalytic H_2_ and H_2_O_2_ (or O_2_) production of Ni_2_P/NiS@PCOS sample without (**a**) and with (**b**) the addition of MnO_2_, **c** H_2_ and O_2_ evolution rate over different samples. **d** H_2_ and O_2_ evolution rate over Ni_2_P/NiS coated with different mole ratio PCOS. **e** Comparison of the overall water splitting activity of Ni_2_P/NiS@PCOS and other typical materials. Experimental conditions: visible-light irradiation (λ > 420 nm), 100 mg photocatalyst, 100 mL deionized water, and 10 mg MnO_2_ assisted by continuous stirring. Error bars indicate standard deviation for three measurements.

Time-dependent photocatalytic overall water splitting performance tests were carried out without adding any sacrificial agent or loading any precious-metal components (Supplementary Fig. 15). As shown in Fig. 3a, the Ni_2_P/NiS@PCOS exhibited excellent hydrogen production activity in the initial 3 hours with a significant reaction decrease subsequently. The oxygen production activity of the material was negligible. In Supplementary Fig. 16a and Supplementary Fig. 17, it was found that the crystal structure and microstructure of Ni_2_P/NiS@PCOS was seriously etched after reaction. Notably, hydrogen peroxide was detected in the reaction solution, and the production rate of H_2_O_2_ is about 100 μmol h^−1^ in the initial 2 h. And that the amount of H_2_ and H_2_O_2_ is not real 1:1. It may explain the poisoning effect of H_2_O_2_ on the photocatalyst^41–43^. As is known, addition of MnO_2_ to the reaction system is an effective method for quickly removing generated H_2_O_2_, thereby preventing poisoning of the photocatalyst^44^. By adding MnO_2_ in the system of reaction, the Ni_2_P/NiS@PCOS showed excellent and stable photocatalytic hydrogen and oxygen production rates, as shown in Fig. 3b. The rate of H_2_ production is 150.7 µmol h^−1^, whereas O_2_ production was measured as 70.2 µmol h^−1^, which is roughly stoichiometric for the overall water splitting reaction, suggesting that the evolved O_2_ is principally originated from the oxidation of water^45^. In addition, the characterization and analysis of the Ni_2_P/NiS@PCOS samples with adding MnO_2_ has been also completed by XRD and XPS tests. The Ni_2_P/NiS@PCOS sample exhibited better structural stability, which was the same as the structural information of samples before the reaction (Supplementary Fig. 16a–f). As shown in Fig. 3c, the activity of Ni_2_P/NiS and Ni_2_P@PCOS are very poor because there are not enough hydrogen production sites and oxygen production sites. However, in the half-reaction tests, the rate of H_2_ production of Ni_2_P/NiS and the rate of O_2_ production of Ni_2_P@PCOS displayed noticeable increase when use of sacrificial agents to scavenge photogenerated holes or electrons, respectively (Supplementary Fig. 18a–d), which powerful proves that the Ni_2_P with NiS is the site for producing H_2_ and the Ni_2_P with PCOS serves as the other site for O_2_ generation. At the same time, it was found that PCOS/NiS and Ni_2_P also have a certain hydrogen or oxygen production capacity in the presence of the sacrificial agent under visible light, demonstrating that PCOS/NiS and Ni_2_P have independent water reduction and oxidation behavior. And the results have a good similarity with other reported of nickel catalyst (Supplementary Table 8). So, when co-loading in Ni_2_P with PCOS and NiS, the as-prepared Ni_2_P/NiS@PCOS sample shows the excellent H_2_ and O_2_ evolution activity, which is about 27 times than that of the Ni_2_P@PCOS and 37.5 folds than the Ni_2_P/NiS. In addition, the performance of Ni_2_P/NiS with varying amounts of PCOS was investigated, with results shown in Fig. 3d. All the Ni_2_P/NiS@PCOS samples showed much superior overall water splitting activity, compared to the pristine Ni_2_P/NiS. The increase in photocatalytic activity was attributed that the PCOS would be changed the electronic structure and reduce the activation energy barrier. And the other series of Ni_2_P/NiS@PCOS with different Ni_2_P and NiS mass ratio were also tested under the same conditions. As shown in Supplementary Fig. 19a and Supplementary Table 2, when the mass ratio of Ni_2_P: NiS is about 5:1, the as-prepared Ni_2_P/NiS@PCOS sample shows the highest activity, which is about 27 times as high as that of the Ni_2_P@PCOS. The increase is attributed to the promotion of charge carrier separation by the NiS cocatalyst.

The optimal Ni_2_P/NiS@PCOS displayed an apparent quantum efficiency of about 7% at 420 nm (Supplementary Fig. 19b) and the solar-to-hydrogen efficiency (STH) was calculated to be as high as 0.91%, indicating that the presence of the dual-site promotes charge separation and the surface redox reaction. In addition, the stability of the Ni_2_P/NiS@PCOS in the photocatalytic overall water splitting was tested in recycling investigations as seen in Supplementary Fig. 19c. The photocatalytic activity of Ni_2_P/NiS@PCOS displayed no noticeable decrease in the reaction period over five consecutive runs, revealing the good stability of this sample. In addition, three independent repeated experiments by three  researchers were performed to further demonstrate the stability and reproducibility of the catalyst (Supplementary Fig. 19e, f). In Supplementary Fig. 19d, the overall water splitting performance of Ni_2_P/NiS@PCOS was also tested at different temperatures (10 °C, 20 °C, 30 °C, 40 °C, 50 °C) with and without light irradiation. Under irradiation conditions, the overall water splitting performance with oxygen production (about ~70 μmol h^−1^) and hydrogen evolution (about ~150 μmol h^−1^) are almost unchanged with the varying of ambient temperatures, but when there is no illumination, the Ni_2_P/NiS@PCOS does not have any activity for producing H_2_ and O_2_. It would be good proof that the temperature is too low for photothermocatalytic synergistic effects to be evident, resulting in little temperature dependence of photocatalytic activity. More importantly, as shown in Fig. 3e and Supplementary Table 6, the overall water-splitting activity of Ni_2_P/NiS@PCOS is a few fold higher than those of supported precious-metal photocatalyst and other benchmarking photocatalysts, indicative of the great advantages of the dual-site with more effective reaction processes.

### Photophysical and electrochemical properties

**Fig. 4:**
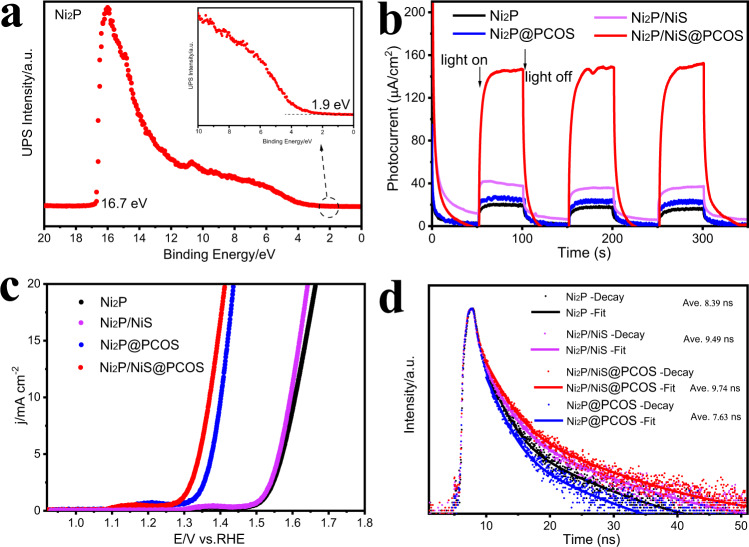
Electrochemical measurements and characterizations of typical samples. **a** UPS spectra of pure Ni_2_P, **b** transient photocurrent density, **c** OER polarization curves, **d** time-resolved fluorescence decay spectra.

As shown in Supplementary Fig. 20, samples of Ni_2_P, NiS, PCOS and Ni_2_P/NiS@PCOS showed broad visible to near-infrared absorption peak, indicating the formation of broadly distributed doping states within the energy gap of Ni_2_P or PCOS^46^. Thus, the UV–Vis spectrum cannot reflect the true forbidden bandwidth. In order to better understand the main factors related to the overall water splitting of the dual-site Ni catalyst, we further investigated the band position of the pure Ni_2_P, NiS, and PCOS by Ultraviolet photoelectron spectra (UPS), Mott–Schottky plots, and Cyclic voltammograms^12,47^. As shown in Fig. 4a, Supplementary Fig. 21 and Supplementary Fig. 22a, b, the UPS of pure Ni_2_P and pure NiS were evaluated with the excitation energy of 21.22 eV (He I). According to the cutoff positions of the second electron, the work functions of Ni_2_P and NiS were calculated to be −4.52 eV and −4.42 eV vs. Vac, respectively (W_f_ = 21.22-E_cutoff_). Therefore, the calculated valence band (VB) edges (*E*_VB_ vs. Vac) of Ni_2_P and NiS are located at 1.92 eV (−6.42 eV vs. Vac) and −0.08 eV (−4.42 eV vs. Vac), respectively (*E*_VB_ = W_f_ + E). The flat-band potentials (*E*_fb_) of NiS and Ni_2_P, were measured to be −0.25 and −0.56 V vs. Ag/ AgCl, respectively, with the conductive band (CB) of Ni_2_P and NiS measured at −0.42 eV (−4.08 eV vs. Vac) and −0.11 eV (−4.39 eV vs. Vac). In addition, the band structure of the PCOS was also analyzed by cyclic voltammetry experiments, as shown in Supplementary Fig. 23, where it was found that the energy band structure of PCOS can be tuned by changing the calcining temperature, summarized in Supplementary Fig. 24 and Supplementary Table 7. In brief, from the view of the band structure, Ni_2_P can achieve water splitting into H_2_ and H_2_O_2_ in theory, coupled with the NiS cocatalyst with the lowest Fermi level (−4.42 eV vs. Vac), which acted as an electron acceptor. The second cocatalyst, PCOS, with lowest valence band acted as a hole donor site to the Ni_2_P via Ni–S–C contribution manifests (Fig. 2g–i), resulting in good charge separation ability due to the synergistic effects of the double cocatalyst. Taking advantage of the synergistic effects of NiS and PCOS, the Ni_2_P/NiS@PCOS photocatalysts exhibited a large number of electron-rich sites and hole-rich sites at the interface of Ni_2_P/NiS and Ni_2_P@PCOS, respectively. And these sites can effectively increase the overall  water splitting activity.

Figure 4b shows the Ni_2_P/NiS@PCOS presented the strongest photocurrent response signals (about 155 μA cm^−2^), which was about 8 and 31 times as high as that of the pure NiS (about 20.0 μA cm^−2^) and Ni_2_P@PCOS (about 5 μA cm^−2^), respectively. Furthermore, the diameter of the Nyquist circle of Ni_2_P/NiS@PCOS (Supplementary Fig. 25) is much smaller than that of pure NiS and Ni_2_P@PCOS. This proved that the Ni_2_P/NiS@PCOS has ultrahigh electron conductivity and mobility. The photoluminescence (PL) testing is a common strategy to investigate the separation efficiency of electron–hole pairs in photocatalysts^30^. In Supplementary Fig. 26, the Ni_2_P/NiS@PCOS sample showed the lowest PL emission peak at the wavelength of 456 nm, when the sample was excited by the wavelength of 325 nm, suggesting that the Ni_2_P/NiS@PCOS possessed a low photogenerated charge carrier recombination rate, showing that the dual-site structure provided a speedy path for electron/hole migration.

Figure 4c shows the electrochemical characterization of Ni_2_P/NiS@PCOS electrodes. It can be seen from the voltage–time curve that the nucleation overpotential of Ni_2_P/NiS@PCOS is lowest (140 mV), while that of Ni_2_P without the PCOS shell is highest (375 mV), the reversible hydrogen electrode (RHE) can be ascribed to H_2_O_2_ evolution from water oxidation occurring at the electrode. In addition, compared to Ni_2_P/NiS and Ni_2_P/NiS, the Ni_2_P/NiS@PCOS and Ni_2_P@PCOS  show a much higher activity and lower overpotential for water oxidation. This result indicates that the PCOS can indeed efficiently electrocatalyze the evolution of O_2_, in good agreement with literature^13^. In Fig. 4d, the time-resolved fluorescence decay spectra further illustrated the advantages of coating PCOS and NiS on the surface of Ni_2_P for the improvement of charge separation. the average lifetime (τ) for Ni_2_P/NiS@PCOS sample are increased, indicating that Ni_2_P with double cocatalyst of NiS and PCOS possesses much longer lifetimes of photogenerated charge carriers than those of Ni_2_P, Ni_2_P@PCOS, and Ni_2_P/NiS, in good accordance with the above analysis.

## Discussion

**Fig. 5:**
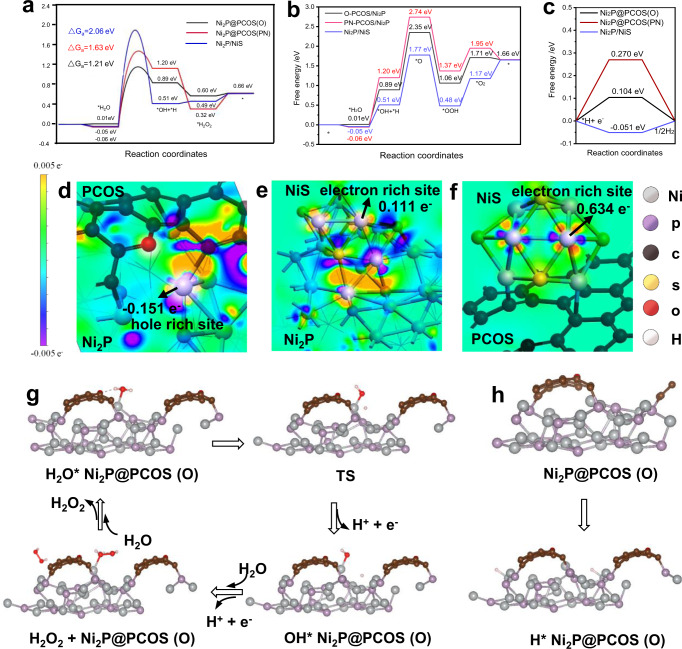
Theoretical study of the promotion effect of the double cocatalyst for overall water splitting. **a**, **b** Gibbs free energy of oxidation reaction path of water molecule on Ni_2_P@PCOS (O site), Ni_2_P@PCOS (pyridine nitrogen site) and Ni_2_P/NiS surface, **c** free-energy barriers for HER, **d**–**f** Charge density difference plots of Ni_2_P@PCOS, Ni_2_P/NiS, NiS/PCOS, respectively. **g** Model diagram of H_2_O_2_ formation by water molecule reaction on Ni_2_P@PCOS (O site). **h** Hydrogen adsorption model diagram on Ni_2_P@PCOS (O site) surface. The TS for transition state. And the yellow, red, pink, violet, brown, and gray spheres represent S, O, H, P, C, and Ni atoms, respectively.

To further understand the relationship between the overall water splitting activity and the synergistic effect between PCOS and NiS of the Ni_2_P/NiS@PCOS catalyst, the density function theory (DFT) calculations was performed. Firstly, we simplify the compound ternary system Ni_2_P/NiS@PCOS into Ni_2_P@PCOS and Ni_2_P/NiS binary systems. Accordingly, the effect of the different main components could be analyzed independently, and the hole-rich and electron-rich Ni sites which contributed to OER and HER process, respectively, could also be described clearly. As is known, the water oxidation half-reaction follows the four-electron reaction pathway for O_2_ generation and the two-electron reaction pathway for H_2_O_2_ generation, which were accompanied by the formation of adsorbed *H_2_O, *OH, *H_2_O_2_, *O, *O_2_, and *OOH intermediates (Fig. 5a, b, g, Supplementary Figs. 29–36, and Supplementary Tables 9–18). We evaluated the possibility that the water adsorption reaction occurred on Ni_2_P/NiS and also on Ni_2_P@PCOS surface, which was considered to be the primary step to influence the photocatalytic reaction^48^. It is worth noting that the doping structure of PCOS is amorphous and hard to combine with the crystalline model as Ni_2_P directly for calculation, thus we simplified the model of PCOS as single-orientation cyclic graphene structure and focus on the effect of hybrid species. The adsorption energy of H_2_O on five hybrid types of C species was used to screen the representative structures and effective reaction sites of PCOS. As shown in Supplementary Table 10, the Ni atoms on interface perform as the main sites with exothermic adsorption of H_2_O, and doping of O and pyridine N atom strengthen the process. The further results of dissociation of *H_2_O was provided in Supplementary Figs. 29–30, which also show the improvement of the dissociation step from the doping of O and pyridine N species. Therefore, the C species with doping of O and N was considered as the active structure of PCOS. Conversely, the S atom, C atom or N atom (graphitic nitrogen) site on the PCOS surface, which displays the Gibbs free energy with much high value, is hard to provide a clear contribution on the OER process and was out of the discussion. Corresponding with the favorable adsorption of H_2_O molecule, the contact-angle measurements provided the results of 34.0–44.5° (Supplementary Fig. 27), which also suggesting a decent hydrophilicity of the surface from experimental results^49^. In the next step, the molecule of water would be dissociated to *OH and *H species, the Gibbs free energies of the transition state are 1.21, 1.63, 2.06 eV for O site on the PCOS surface, the N (pyridine nitrogen) site on the PCOS surface, and the NiS site, respectively. For the O site on the PCOS surface, the Gibbs free energies of each the elementary steps were the lowest, and its rate-limiting step was substantially available to complete the reaction. Confirmation of the *OH species was determined experimentally by the spin trapping reagent of TEMPO, shown in Supplementary Fig. 28. In the third step, there are two different reaction pathways for further water oxidation (H_2_O_2_ or O_2_) from *OH (Fig. 5a, b, g). It was found, at the Ni_2_P@PCOS(O site) surface, the route of *H_2_O_2_ is more favorable by thermodynamic spontaneous step with exothermic free-energy change, while a much higher energy change of 1.46 eV is required for the route of *O. Therefore, H_2_O_2_ is preferentially formed at the surface of Ni_2_P@PCOS (O site). Additionally, in the four-electron reaction pathway, the *O species still reacted with another *H_2_O to form the OOH* species with a ΔG of −1.29 eV, and the OOH* species released electron and proton to form the O_2_, which is an endothermic process with a ΔG of 0.65 eV (Fig. 5b, Supplementary Fig. 34). As a comparison, we also compared the reaction pathways for further water oxidation (H_2_O_2_ or O_2_) from *OH at the Ni_2_P@PCOS(PN site) surface and Ni_2_P/NiS surface, the hydrogen peroxide production path was also more favorable (Fig. 5b and Supplementary Figs. 32, 33, 35, 36). These comparative results indicate that the Ni_2_P@PCOS and Ni_2_P/NiS preferentially produced H_2_O_2_ via a two-step/two-electron pathway instead of producing O_2_ through one-step/four-electron. Notably, we also note that the concentration of positive charge on Ni_2_P is very high, because a lot of hole transfer occurs from the PCOS region to the Ni_2_P, which would be conducive to carrying out the oxidation reaction (Fig. 5d). Thus, the hole-rich Ni_2_P with PCOS is the main site for H_2_O_2_ generation, which agrees well with the observations in experimental results.

As for the HER process, the generated H* would combine to form H_2_ and the free energy of hydrogen adsorption (ΔG_H*_) on the surface of photocatalyst was often used as a descriptor for HER. As we all know, the ΔG_H*_ of an ideal HER catalyst should be close to 0, which indicates that the catalysts are hard to poison. For Ni_2_P@PCOS, the high positive ΔG_H*_ of the O site and PN sites (0.27 eV and 0.10 eV) suggest that the HER on the PCOS surface is nonspontaneous and needs to absorb a lot of energy to initiate^50^. However, it was discovered that the hydrogen-binding energies on the top of NiS site by −0.051 eV are much thermo-neutral and almost as efficient as Pt for the evolution of H_2_ (in Fig. 5c, h and Supplementary Figs. 32–33)^51^. In addition, it is obvious that Ni atom of NiS owns more negative charge because a lot of electron transfer occurs from the Ni_2_P and PCOS region to the NiS (Fig. 5e, f).

**Fig. 6:**
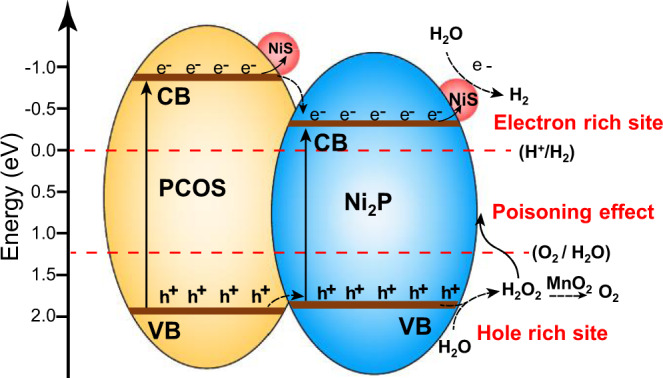
Scheme illustrating the principle of charge transfer and the photocatalytic processes for overall water splitting on the interface of the Ni_2_P/NiS@PCOS under visible-light irradiation. Electron-rich sites: the interface of Ni_2_P/NiS. Hole-rich sites: the interface of Ni_2_P@PCOS.

The charge distribution of Ni_2_P@PCOS, Ni_2_P/NiS and NiS/PCOS surface was provided in Fig. 5d–f to verify the existence of two types of Ni species (hole rich of Ni site (Ni_2_P) and electron-rich of Ni site (NiS)). We can see that the prominent electron transfer occurs from the Ni_2_P and PCOS region to the NiS, which benefits for the electron-rich of Ni site (NiS). In addition, it is obvious observed that Ni atom of Ni_2_P owns more positive charges because the hole transfer occurs from the PCOS region to the Ni_2_P. Based on the above characterizations and analysis, the proposed mechanisms for Ni_2_P/NiS@PCOS photocatalyst in the overall water splitting process can be described as follows (as shown in Fig. 6). Under visible-light irradiation, numerous electron–hole pairs are first generated in the surface of Ni_2_P and PCOS. Then, the NiS could efficiently capture the photoexcited electrons from the Ni_2_P or PCOS, the interface of Ni_2_P/NiS act as the electron-rich sites for reducing H_2_O into H_2_. Many of the photoexcited holes left in the valence band of Ni_2_P or also accumulate from the hole donor of PCOS to Ni_2_P surface. Thus, the interface of Ni_2_P@PCOS would act as the hole-rich sites for oxidizing H_2_O into OH radicals (2.38 V vs. RHE)^52^, and then two OH radicals combine to form one H_2_O_2_, which would be decomposed O_2_ and H_2_O when adding any MnO_2_ in the system.

In summary, this work presents a good idea in the design of high-performance overall water splitting photocatalysts by selecting a simpler kinetic pathway for water oxidation, effectively reducing the thermodynamic energy barrier. We designed an electron–hole rich dual-site photocatalyst (Ni_2_P/NiS@PCOS) for overall water splitting under visible-light irradiation. The obtained Ni_2_P/NiS@PCOS photocatalyst exhibited overall water splitting with H_2_ and O_2_ production at nearly 2:1 ratio. It was discovered that Ni_2_P/NiS@PCOS can provide a simple reaction pathway of two-step/two-electron process, resulting in the selective water oxidation pathway. And the PCOS and NiS-induced variation in electron–hole rich state of Ni atom effectively optimized thermodynamics of water dissociation and accelerated reaction kinetics for electron migration. The electron–hole rich state of nickel catalyst controls the catalytic activity by virtue of tuning the electronic structure. Such atomic-level design of the structure–activity interactions can more understand and guide other catalytic reactions.

## Methods

### Catalyst preparation

#### Preparation of the NiS@l-cysteine nanosheets

The NiS@l-cysteine nanosheets were prepared by a one-step hydrothermal process. In this procedure, 2 mmol NiCl_2_ and 2 mmol l-cysteine are mixed in 25 mL deionized water, and continuously stirring 6 h. Then, 10 mL solution with a certain concentration Na_2_S was injected into above solution. After continuously stirring 10 h, the mixed solution was then transferred into a Teflon-lined stainless-steel autoclave and heated at 110 °C for 2 h. After filtration and washing with deionized water several times, the resulting precipitant was dried using a vacuum freeze dryer overnight to finally obtain NiS@l-cysteine nanosheets.

#### Preparation of Ni_2_P/NiS@PCOS

In all, 0.1 g NiS@l-cysteine nanosheets and sodium hypophosphite hydrate powder were respectively placed in the both end of a rectangle porcelain boat. And then the porcelain boat was tightly packaged with aluminum foil and put into a tube furnace. The furnace was charged with nitrogen as the carrying gas before being turned on, which was maintained at a flow rate of 20 mL/min, and the furnace reached the desired temperature (350 °C) with a constant ramp rate of 5 °C/min. After 180 min reaction, the Ni_2_P/NiS@l-cysteine heterojunction was obtained. The samples prepared with different sodium hypophosphite powder amount of 0 g, 0.1 g, 0.3 g, 0.5 g, 0.7 g, and 0.9 g were denoted as NiS@PCOS, Ni_2_P/NiS@PCOS-1, Ni_2_P/NiS@PCOS-3, Ni_2_P/NiS@PCOS-5 (or denoted as Ni_2_P/NiS@PCOS), Ni_2_P/NiS@PCOS-7, and Ni_2_P/NiS@PCOS-9 (or denoted as Ni_2_P@PCOS), respectively.

#### Preparation of PCOS

In a quartz tube, 1 g l-cysteine powder was put in the middle area of furnace. Nitrogen as the carrying gas was then filled into the quartz tube before the tube furnace was turned on. The furnace was charged with nitrogen as the carrying gas before being turned on, which was maintained at a flow rate of 20 mL/min, and the furnace reached the desired temperature (350–450 °C) with a constant ramp rate of 5 °C/min. After 180 min reaction, the PCOS was obtained. The samples prepared with different temperature of 300 °C, 350 °C, 400 °C, and and 450 °C were denoted as PCOS_300_, PCOS_350_, PCOS_400_, and PCOS_450_, respectively.

#### Preparation of Ni_2_P/NiS coated with different mole ratio PCOS

Overall, 0.1 g pure NiS (without coated with l-cysteine by a one-step hydrothermal process) and a certain amount of l-cysteine are mixed in 1 mL deionized water, and then dried at 60 °C for 3 h. Finally, the precipitate was phosphating under the same conditions as above. The samples prepared with different mole ratio of 0%, 5%, and 10% l-cysteine were denoted as Ni_2_P/NiS@0%PCOS, Ni_2_P/NiS@5%PCOS, and Ni_2_P/NiS@10%PCOS, respectively.

### Catalyst characterization

X-ray powder diffraction (SHIMADZU Lab X XRD-6100) was used to analyzed the crystal phases of all as-prepared photocatalysts, and with a 2*θ* range scanned from 10 to 80° at a rate of 10° min^−1^. The surface and inner structure information of the materials were obtained by a field-emission scanning electron microscope (SEM, JEOL, JSM-6700F) and transmission electron microscopy (TEM, JEOL, JEM-2100), respectively. X-ray photoelectron spectroscopy (XPS) was tested to investigate the chemical valence states of the samples (Kratos AXIS Ultra Dld photoelectron spectrometer with monochromatic Al-Ka excitation), and the binding energies were also calibrated using the C 1 s internal standard peak at 284.6 eV. The carrier separation ability and fluorescence lifetime were tested by a FLS980 fluorescence spectrophotometer (Edinburgh instruments) and the electron paramagnetic resonance was measured using a Bruker Biospin A300 spectrometer. The information of function groups was recorded by using Raman measurements (thermo Scientific DXR3 xi) and Fourier transform infrared spectra (Nicolet avatar 360). In addition, the optical adoption properties was obtained by UV–vis diffuse reflection spectra (UV–vis DRS, SHIMADZU, UV-2600). Solid-state ^13^C-nuclear magnetic resonance spectra was tested by a solid-state NMR spectrometer (Bruker Avance III 400 MHz). And the specific surface area and pore size distribution plots were measured by nitrogen adsorption–desorption apparatus (Brunauer–Emmett–Teller, BELsorp).

### XAFS tested

The X-ray absorption fine structure spectra (XAFS) Ni K–edge were collected at BL07A1 beamline of National Synchrotron Radiation Research Center (NSRRC). The data were collected in fluorescence mode using a Lytle detector while the corresponding reference sample were collected in transmission mode. The sample were grinded and uniformly daubed on the special adhesive tape. The obtained XAFS data was processed in Athena (version 0.9.26) for background, pre-edge line and post-edge line calibrations. Then Fourier transformed fitting was carried out in Artemis (version 0.9.26). The k^3^ weighting, k-range of 3−14 Å^−1^ and R range of 1–3 Å were used for the fitting of Co foil; k-range of 3−11 Å^−1^ and R range of 1–2 Å were used for the fitting of samples. The four parameters, coordination number, bond length, Debye–Waller factor and E_0_ shift (CN, R, ΔE_0_) were fitted without anyone was fixed, the σ^2^ was set. For Wavelet Transform analysis, the χ(k) exported from Athena was imported into the Hama Fortran code. The parameters were listed as follow: R range, 1−4 Å, k-range, 0−12 Å^−1^ for samples; k weight, 3; and Morlet function with κ = 10, σ = 1 was used as the mother wavelet to provide the overall distribution.

### Computational methods

All the computational calculations were adopted using the Vienna ab initio simulation package (VASP). The electron ion interaction was described using the projector-augmented wave (PAW) approach. The exchange-correlation term was described by the Perdew Burke Ernzerhof (PBE) functional. In addition, the van der Waals interactions were described using the empirical correction in Grimme’s scheme (DFT + D2). Cutoff over 400 eV for the plane wave expansion was adopted using 400 eV in all the calculations. A Monkhorst–Pack 3 × 2 × 1 k-point grid was used with a vacuum space of 25 Å. When the total energy changes were less than 1 × 10^−5 ^eV per atom and the Hellmann–Feynman force on each atomic site was within 0.05 eV/Å, the structure optimization calculations were done. Ni_2_P lattice model with space group P-62m is used and (111) surface of Ni_2_P is adopted as reaction plane. For NiS/Ni_2_P system, we build NiS cluster on the Ni_2_P (111) surface as heterojunction system. For PCOS/Ni_2_P system, a unidirectional cycled graphene layer with 18 carbon atoms was built on the Ni_2_P (111)  surface for provide the sites on interface, and the atom coordinated with Ni atom was replaced with O, N, S respectively for descripting the effect of different species in PCOS. The structure models were provided in the accessory, and the calculation parameters were also given with form of INCAR file.

The Gibbs free-energy values (∆G) for the hydrogen reduction and water oxidation reactions were referenced to the computational hydrogen electrode model, utilizing the proton-coupled electron transfer method. The chemical potential of the H^+^/e^–^ pair was considered as half of the H_2_ gas molecule in this model. The ∆G value was determined using the formula: ∆G = ∆E + ∆ZPE – T∆S, where ∆E is the reaction energy difference between the product and reactant occurring on the catalysts. The adsorption energy (E_ads_) was calculated using the formula: E_ads_ = E_total_ − E_substrate_ − E_adsorbate_, where E_adsorbate_, E_substrate_, and E_total_ represent the substrate, the adsorbate, and the total energies of the system containing the substrate and adsorbate, respectively. The entropies and frequencies of the molecules in the gas phase are taken from the NIST database. The free energy of O_2_(g) was derived as G_O2_ = 2G_H2O_ − 2G_H2_ − 4.92 eV since O_2_ in triplet ground state is notoriously poorly described by DFT calculations. The Gibbs free energy of H^*^ (ΔG_H*_) was calculated by the equation ΔG_H*_ = ΔE_H*_ + ΔZPE − TΔS, where ΔE_H*_, ΔZPE, and ΔS are the adsorption energy, zero point energy, and entropy change. ΔZPE was obtained by ΔZPE = ZPE (H^*^) − 1/2ZPE (H_2_), and in special, ΔS was obtained by ΔS = S(H^*^) − 1/2 S(H_2_) ≈ −1/2 S(H_2_) because of negligible vibrational entropy of H*. At 300 K and 1 atm, TS(H_2_) = 0.41 eV, thus TΔS = −0.205 eV. The detailed process for analysis of data was provided in Supplementary Tables 9–16.

### Evaluation of photocatalytic activity

#### Photocatalytic H_2_ and O_2_ evolution

The photocatalytic H_2_ and O_2_ production reaction from water was carried out in a Labsolar-6A all-glass automatic inline trace gas analysis system, the chromatographic carrier gas was argon. Typically, 100 mg photocatalyst was dispersed in 100 mL aqueous solution. For H_2_ and O_2_ production experiments, the reaction vessel also contained 10 mg MnO_2_. The light is irradiated from the top of the reaction cell. The solution was degassed by vacuum-drawing for 10 min to remove the O_2_ in the reaction cell (the vacuum pressure is 13 kpa) and then irradiated under visible light (>420 nm) at room temperature. Finally, the amount of H_2_ and O_2_ production was analyzed by on line gas chromatography (Beifen-Ruili, SP-2100A, China).

The apparent quantum efficiency (AQE) of photocatalytic H_2_ production was tested under similar conditions. A Xe lamp (300 W), as the light source, was equipped with a 420/475/550 nm bandpass filter. And the amount of H_2_ evolution was counted after reacted for 3 h. The AQE for H_2_ evolution was calculated according to the following equation and the other parameters are shown in Supplementary Fig. 19.

1 $${{\mbox{AQE}}}\left( \% \right) \,=	\frac{{{{{{\rm{number}}}}}}\,{{{{{\rm{of}}}}}}\,{{{{{\rm{reacted}}}}}}\,{{{{{\rm{electrons}}}}}}}{{{{{{\rm{number}}}}}}\,{{{{{\rm{of}}}}}}\,{{{{{\rm{incident}}}}}}\,{{{{{\rm{photons}}}}}}} \times 100 \% \\=	\frac{2 \times {{{{{\rm{number}}}}}}\,{{{{{\rm{of}}}}}}\,{{{{{\rm{evolved}}}}}}\,{{{{{\rm{hydrogen}}}}}}\,{{{{{\rm{molecules}}}}}}}{{{{{{\rm{number}}}}}}\,{{{{{\rm{of}}}}}}\,{{{{{\rm{incident}}}}}}\,{{{{{\rm{photons}}}}}}} \times 100 \% \\=	\frac{(v{{\times }}{N}_{A}\times 2){{\times }}(h{{\times }}c)}{(I{{\times }}A{{\times }}\lambda )} \times 100 \% .$$

The STH was evaluated using a 300 W Xe lamp with a total reflection filter and an AM1.5 G filter (100 mW cm^−2^) as the simulated solar light source. In order to improve illumination uniformity and reduce errors, the irradiation spot area that light put into the reactor is adjusted to 10 cm^2^. The amount of H_2_ evolution was counted after reacting for 3 h. The STH was calculated according to following equation, and the other parameters are shown in Supplementary Fig. 20.

2 $${{{{{\rm{STH}}}}}}\,\left( \% \right)=\frac{{{{{{\rm{Energy}}}}}}\,{{{{{\rm{of}}}}}}\,{{{{{\rm{generated}}}}}}\,{{{{{{\rm{H}}}}}}}_{2}}{{{{{{\rm{Solar}}}}}}\,{{{{{\rm{energy}}}}}}\,{{{{{\rm{irradiating}}}}}}\,{{{{{\rm{the}}}}}}\,{{{{{\rm{reactor}}}}}}}{{\times }}100 \%=\frac{{R}_{{H}_{2}}{{\times }}\triangle {G}_{r}}{{P}_{{sun}}{{\times }}S}{{\times }}100 \% .$$

#### Photoelectrochemical measurement

Electrochemical impedance spectroscopy (EIS) and transient photocurrent response of samples was performed on an electrochemical analyzer (PMC-1000/DC, AMETEK) equipped with a standard three-electrode system. In this system, 1 M Na_2_SO_4_ aqueous solution served as the electrolyte. A platinum wire, an Ag/AgCl electrode and glassy carbon electrodes coated with obtained samples were chosen as the counter electrode, reference electrode, and working electrodes, respectively.

#### Hydrogen peroxide detection

The formation of H_2_O_2_ was detected by UV–vis spectroscopy with o-tolidine as the peroxide indicator^53^. In brief, 2 mL of the suspension was centrifuged (11,180 × *g*) to remove the photocatalyst, which could be able to suppress the UV absorption background. Then, 0.5 mL 1% o-tolidine in HCl solution (0.1 M) was added into above suspension. Next, the suspension was acidified with 2 mL HCl (1 M). Finally, the amount of H_2_O_2_ generated was assessed by UV–vis spectroscopy and the characteristic maximum peak at 436 nm.

## Supplementary information

Supporting Information

## Data Availability

The data that support the findings of this study are included in the published article (and its Supplementary Information) or available from the corresponding author on reasonable request.  Source data are provided with this paper.

## Source data

Source Data

## References

[1] Wang Z, Li C, Domen K (2019). Recent developments in heterogeneous photocatalysts for solar-driven overall water splitting. Chem. Soc. Rev..

[2] Chen S, Takata T, Domen K (2017). Particulate photocatalysts for overall water splitting. Nat. Rev. Mater..

[3] Kudo A, Miseki Y (2009). Heterogeneous photocatalyst materials for water splitting. Chem. Soc. Rev..

[4] Xu JQ (2019). Visible‐light‐driven overall water splitting boosted by tetrahedrally coordinated blende cobalt(II) oxide atomic layers. Angew. Chem. Int. Ed..

[5] Cui XK (2018). Insights into highly improved solar-driven photocatalytic oxygen evolution over integrated Ag_3_PO_4_/Mos_2_ heterostructures. Front. Chem..

[6] Meng QG, Yang JJ, Ma SX, Zhai MJ, Lu JT (2017). A porous cobalt (II) metal–organic framework with highly efficient electrocatalytic activity for the oxygen evolution reaction. Polymers.

[7] Zhao Y, Nakamura R, Kamiya K, Nakanishi S, Hashimoto K (2013). Nitrogen-doped carbon nanomaterials as non-metal electrocatalysts for water oxidation. Nat. Commun..

[8] Ma TY, Dai S, Jaroniec M, Qiao SZ (2014). Graphitic carbon nitride nanosheet–carbon nanotube three-dimensional porous composites as high-performance oxygen evolution electrocatalysts. Angew. Chem. Int. Ed..

[9] Kim HI, Kwon OS, Kim S, Choi W, Kim JH (2016). Harnessing low energy photons (635 nm) for the production of H_2_O_2_ using upconversion nanohybrid photocatalysts. Energy Environ. Sci..

[10] Srinivasan N, Sakai E, Miyauchi M (2016). Balanced excitation between two semiconductors in bulk heterojunction Z-scheme system for overall water splitting. Acs. Catal..

[11] Takanabe K (2017). Photocatalytic water splitting: quantitative approaches toward photocatalyst by design. Acs. Catal..

[12] Wu XQ (2017). Control strategy on two-/four-electron pathway of water splitting by multidoped carbon based catalysts. Acs. Catal..

[13] Hou Y (2015). An advanced nitrogen-doped graphene/cobalt-embedded porous carbon polyhedron hybrid for efficient catalysis of oxygen reduction and water splitting. Adv. Funct. Mater..

[14] Pan ZM, Zheng Y, Guo FS, Niu PP, Wang XC (2017). Decorating Cop and Pt nanoparticles on graphitic carbon nitride nanosheets to promote overall water splitting by conjugated polymers. Chemsuschem.

[15] Li JY (2016). Mechanistic insights on ternary Ni_2−x_Co_x_P for hydrogen evolution and their hybrids with graphene as highly efficient and robust catalysts for overall water splitting. Adv. Funct. Mater..

[16] Bruix A (2012). A new type of strong metal–support interaction and the production of H_2_ through the transformation of water on Pt/CeO_2_ (111) and Pt/CeOx/TiO_2_ (110) catalysts. J. Am. Chem. Soc..

[17] Campbell CT (2012). Electronic perturbations. Nat. Chem..

[18] Lykhach, Y. et al. Counting electrons on supported nanoparticles. *Nat. Mater.* **15**, 284–288 (2016). 10.1038/nmat4500 26657332

[19] Shi Y (2021). Electronic metal–support interaction modulates single-atom platinum catalysis for hydrogen evolution reaction. Nat. Commun..

[20] Tian B, Li Z, Zhen WL, Lu GX (2016). Uniformly sized (112) facet Co_2_P on graphene for highly effective photocatalytic hydrogen evolution. J. Phys. Chem. C..

[21] Wen LL (2019). Flexible vanadium-doped Ni_2_P nanosheet arrays grown on carbon cloth for an efficient hydrogen evolution reaction. Nanoscale.

[22] Kurman Y, Kaminer I (2020). Tunable bandgap renormalization by nonlocal ultra-strong coupling in nanophotonics. Nat. Phys..

[23] Zhao FL (2020). Two-dimensional gersiloxenes with tunable bandgap for photocatalytic H_2_ evolution and CO_2_ photoreduction to CO. Nat. Commun..

[24] Yan XQ (2018). Fabrication of novel all-solid-state Z-scheme heterojunctions of 3DOM-WO_3_/Pt coated by mono-or few-layered WS_2_ for efficient photocatalytic decomposition performance in Vis-NIR region. Appl. Catal. B-Environ..

[25] Ning CZ, Dou LT, Yang PD (2017). Bandgap engineering in semiconductor alloy nanomaterials with widely tunable compositions. Nat. Rev. Mater..

[26] Liu Y (2021). Charge storage of carbon dot enhances photo-production of H_2_ and H_2_O_2_ over Ni_2_P/carbon dot catalyst under normal pressure. Chem. Eng. J..

[27] Li SH, Zhang N, Xie XQ, Luque R, Xu YJ (2018). Stress-transfer-induced in situ formation of ultrathin nickel phosphide nanosheets for efficient hydrogen. Evol. Angew. Chem. Int. Ed..

[28] Xue ZH (2017). Janus Co/CoP nanoparticles as efficient Mott–Schottky Electrocatalysts for overall water splitting in wide pH range. Adv. Energy Mater..

[29] Lia YB, Jin ZL, Wang HY, Zhang YP, Liu H (2019). Effect of electron-hole separation in MoO_3_@Ni_2_P hybrid nanocomposite as highly efficient metal-free photocatalyst for H_2_ production. J. Colloid Interf. Sci..

[30] Xue C (2018). Nisx quantum dots accelerate electron transfer in Cd0.8Zn0.2S photocatalytic system via an rGO nanosheet “bridge” toward visible-light-driven hydrogen evolution. Acs. Catal..

[31] Qiu HJ (2015). Nanoporous graphene with single-atom nickel dopants: an efficient and stable catalyst for electrochemical hydrogen production. Angew. Chem. Int. Ed..

[32] Liu ZQ, Cheng H, Li N, Ma TY, Su YZ (2016). ZnCo_2_O_4_ quantum dots anchored on nitrogen-doped carbon nanotubes as reversible oxygen reduction/evolution electrocatalysts. Adv. Mater..

[33] Robel I, Subramanian V, Kuno M, Kamat PV (2006). Quantum dot solar cells. Harvesting light energy with CdSe nanocrystals molecularly linked to mesoscopic TiO_2_ films. J. Am. Chem. Soc..

[34] Shen YJ, Lee YL, Yang YM (2006). Monolayer behavior and Langmuir−Blodgett manipulation of CdS quantum dots. J. Phys. Chem. B..

[35] Shi R (2018). Interstitial P-doped CdS with long-lived photogenerated electrons for photocatalytic water splitting without sacrificial agents. Adv. Mater..

[36] Qu KG (2017). Polydopamine-inspired, dual heteroatom-doped carbon nanotubes for highly efficient overall water splitting. Adv. Energy Mater..

[37] Chandra, S. et al. Luminescent S-doped carbon dots: an emergent architecture for multimodal applications. *J. Mater. Chem. B.* **1**, 2375–2382 (2013). 10.1039/c3tb00583f 32261072

[38] Du ZM (2018). C_60_-decorated nickel–cobalt phosphide as an efficient and robust electrocatalyst for hydrogen evolution reaction. Nanoscale.

[39] Bulusheva LG (2011). Electrochemical properties of nitrogen-doped carbon nanotube anode in Li-ion batteries. Carbon.

[40] Wang F (2016). A “solid dual-ions-transformation” route to S,N Co-doped carbon nanotubes as highly efficient “metal-free” catalysts for organic reactions. Adv. Mater..

[41] Liu J (2015). Metal-free efficient photocatalyst for stable visible water splitting via a two-electron pathway. Science.

[42] Mateo D (2016). 111 Oriented gold nanoplatelets on multilayer graphene as visible light photocatalyst for overall water splitting. Nat. Commun..

[43] Hisatomi T, Kubota J, Domen K (2014). Recent advances in semiconductors for photocatalytic and photoelectrochemical water splitting. Chem. Soc. Rev..

[44] Rusek JJ (1996). New decomposition catalysts and characterization techniques for rocket-grade hydrogen peroxide. J. Propul. Power.

[45] Zhao DM (2021). Boron-doped nitrogen-deficient carbon nitride-based Z-scheme heterostructures for photocatalytic overall water splitting. Nat. Energy.

[46] Hou Y (2017). A generic interface to reduce the efficiency-stability-cost gap of perovskite solar cells. Science.

[47] Xue C, Zhang P, Shao GS, Yang GD (2020). Effective promotion of spacial charge separation in direct Z-scheme WO_3_/CdS/WS_2_ tandem heterojunction with enhanced visible-light-driven photocatalytic H_2_ evolution. Chem. Eng. J..

[48] Lin B, Yang GD, Wang LZ (2019). Stacking-layer-number dependence of water adsorption in 3D ordered close-packed g-C_3_N_4_ nanosphere arrays for photocatalytic hydrogen. Evol. Angew. Chem. Int. Ed..

[49] He YL (2018). Hydrophobic CuO nanosheets functionalized with organic adsorbates. J. Am. Chem. Soc..

[50] Xu BB (2022). Synergistic promotion of single-atom co surrounding a PtCo alloy based on a g‑C_3_N_4_ nanosheet for overall water splitting. ACS Catal..

[51] Chen H (2016). Promoting subordinate, efficient ruthenium sites with interstitial silicon for Pt‐like electrocatalytic activity. Angew. Chem. Int. Ed..

[52] Wang Y (2020). Unique hole-accepting carbon-dots promoting selective carbon dioxide reduction nearly 100% to methanol by pure water. Nat. Commun..

[53] Fu YJ (2018). Photocatalytic H_2_O_2_ and H_2_ generation from living *Chlorella vulgaris* and carbon micro particle comodified g-C_3_N_4_. Adv. Energy Mater..

