# Supplementary material for: An electron-hole rich dual-site nickel catalyst for efficient photocatalytic overall water splitting
Source: Nat Commun. 2023 Mar 29;14:1741. doi: 10.1038/s41467-023-37358-3 (PMC10060254; doi:10.1038/s41467-023-37358-3)
Supplement: Supplementary file 1 — Supporting Information [file 41467_2023_37358_MOESM1_ESM.pdf]

Supporting Information

An Electron-Hole Rich Dual-Site Nickel Catalyst for Efficient Photocatalytic Overall Water Splitting

Xiaoqing Yan[1], Mengyang Xia[1], Hanxuan Liu[1], Bin Zhang[2], Chunran Chang[1], Lianzhou Wang[3], Guidong Yang[1]*

[1] School of Chemical Engineering and Technology, Xi'an Jiaotong University, Xi'an 710049, P.R. China

[2] College of Physics and Optoelectronic Engineering, Shenzhen University, Shenzhen 518060, P.R. China

[3] School of Chemical Engineering and Australian Institute for Bioengineering and Nanotechnology, the University of Queensland, QLD, 4072, Australia

Corresponding Author *E-mail: guidongyang@xjtu.edu.cn

This PDF file includes:

Supplementary Figures 1 to 36

Supplementary Tables 1 to 20

Supplementary Notes 1 to 3

Supplementary References

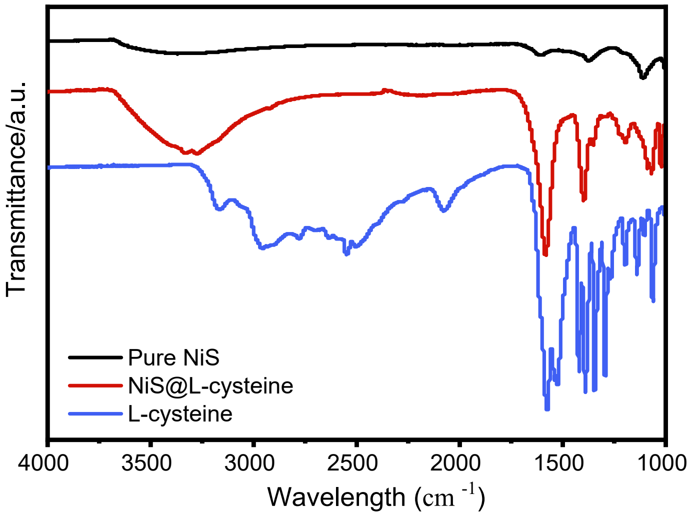

Supplementary Figure 1. FT-IR spectra of pure NiS nanosheets, NiS@L-cysteine and pure L-cysteine.

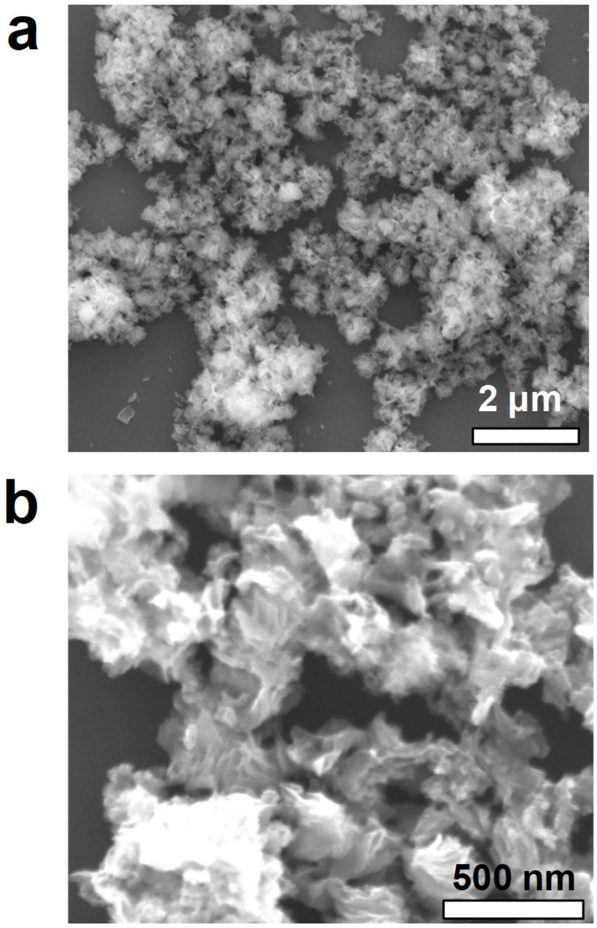

Supplementary Figure 2. (a, b) SEM images of the NiS@ L-cysteine.

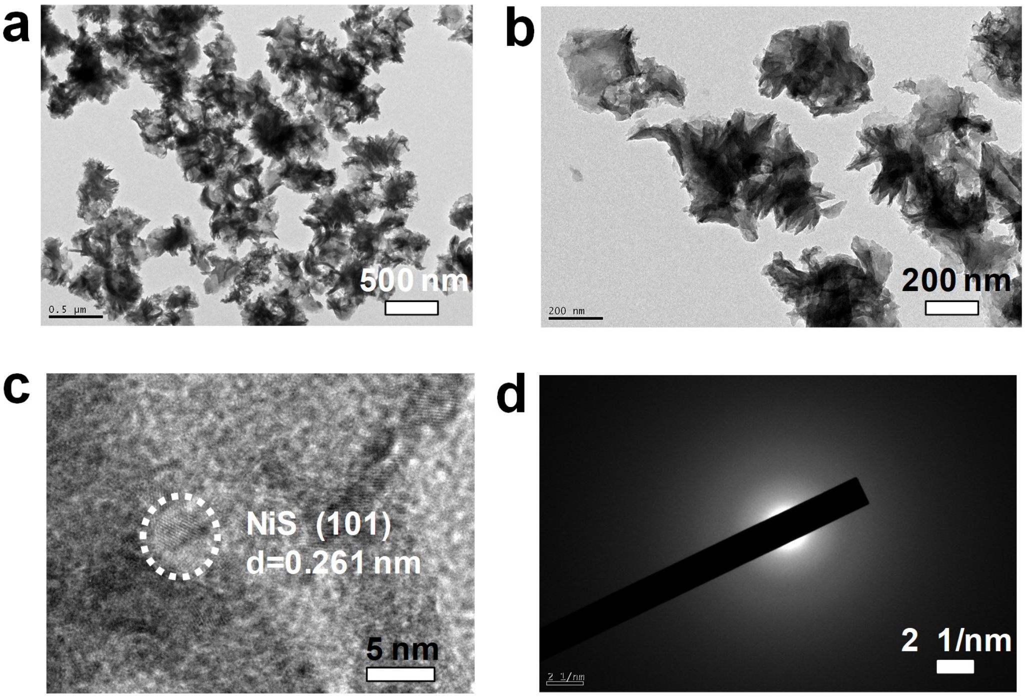

Supplementary Figure 3. TEM(a, b), HRTEM (c) and the selected-area electron diffraction (d) images of the NiS @L-cysteine.

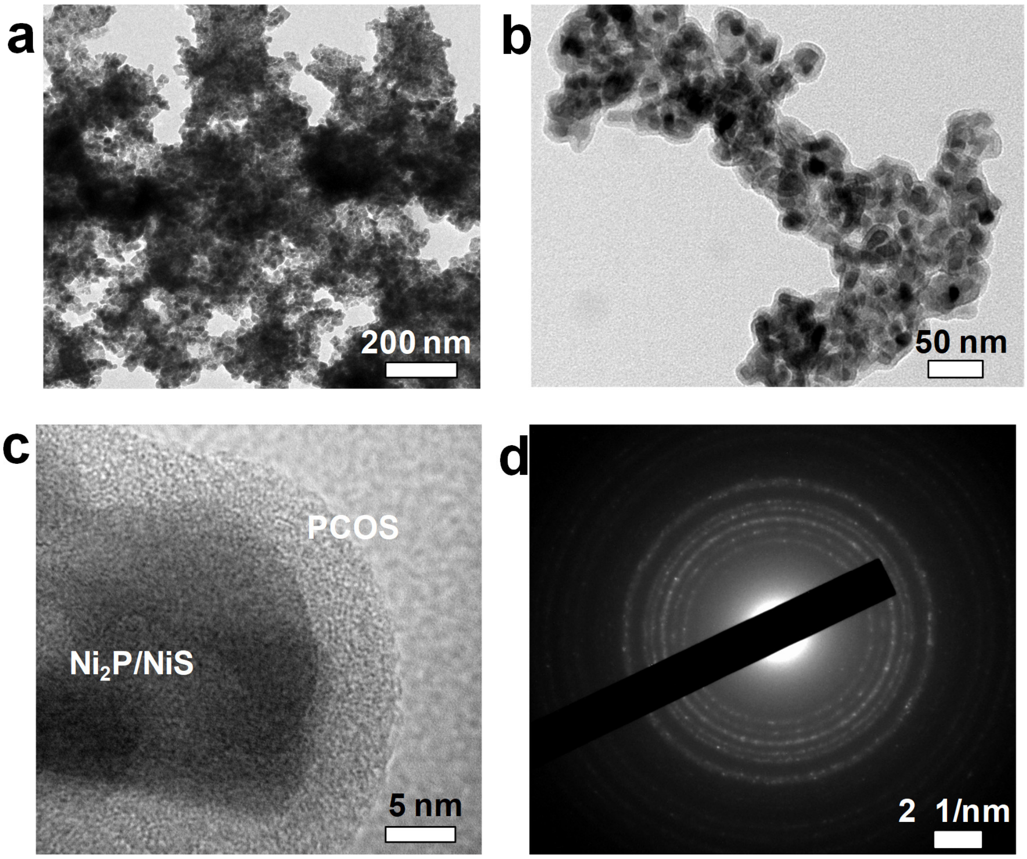

Supplementary Figure 4. (a-c) TEM images of the Ni_2P/NiS@PCOS. (d) the selected-area electron diffraction pattern of Ni_2P/NiS@PCOS.

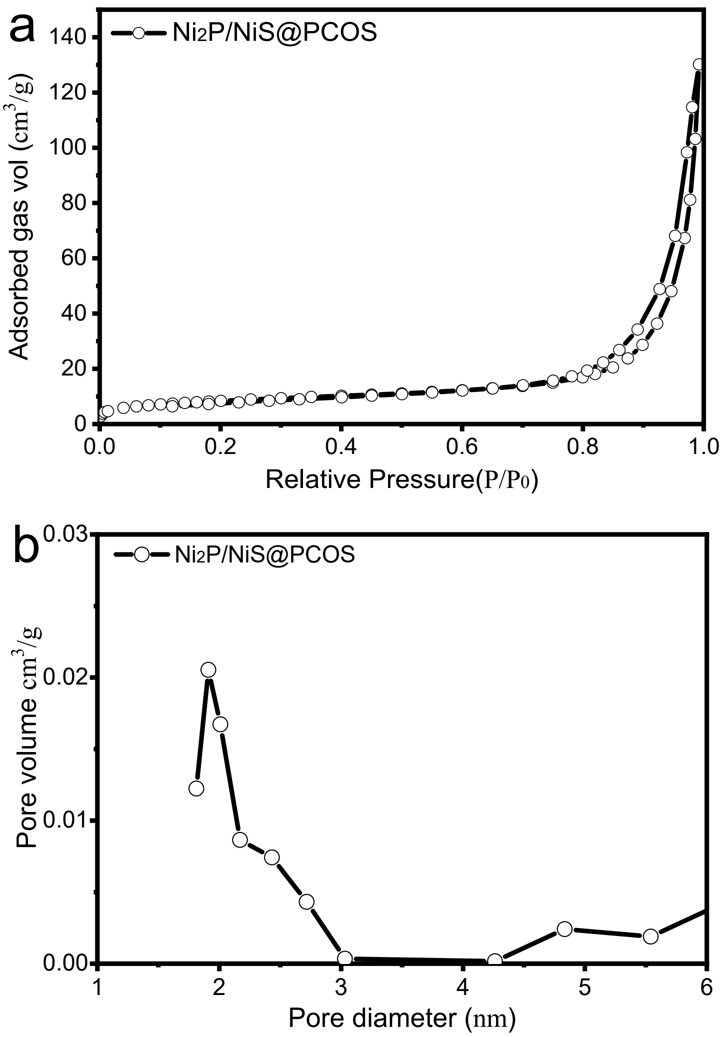

Supplementary Figure 5. (a) Nitrogen adsorption-desorption isotherms and (b) the corresponding pore size distributions curve of $Ni_2P/NiS@PCOS$ sample.

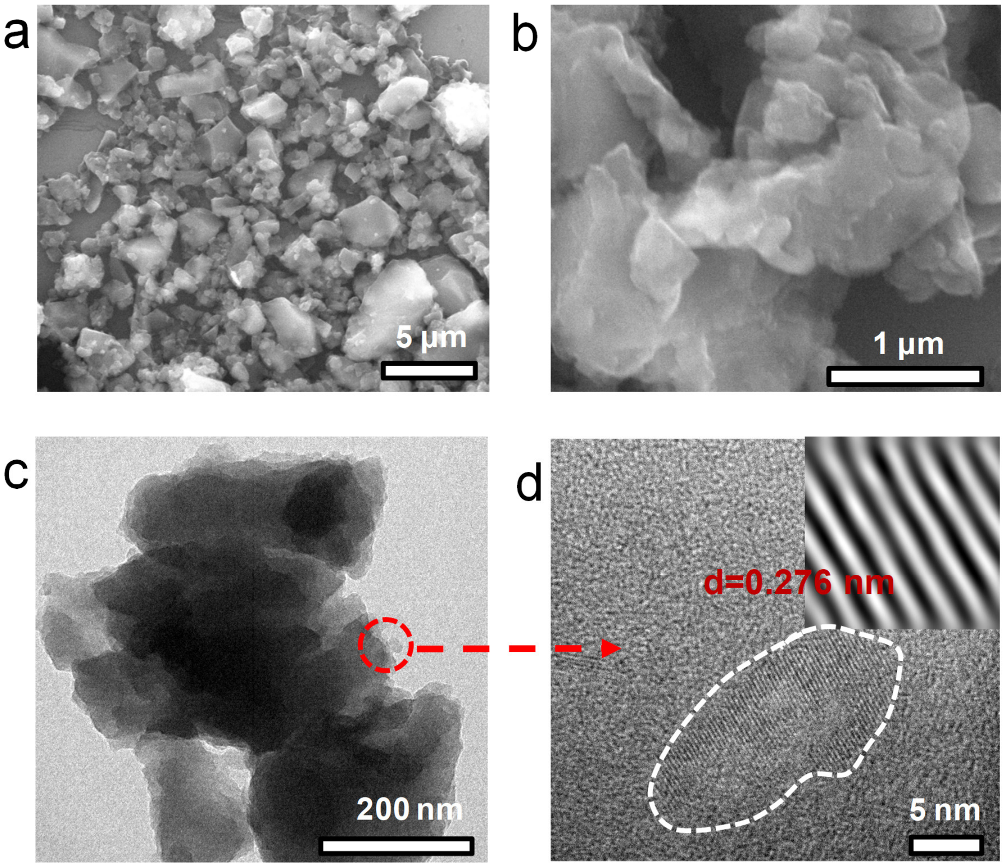

Supplementary Figure 6. (a-b) SEM and (c-d) TEM/HRTEM images of the polymeric carbon-oxygen semiconductor ($PCOS_{350}$).

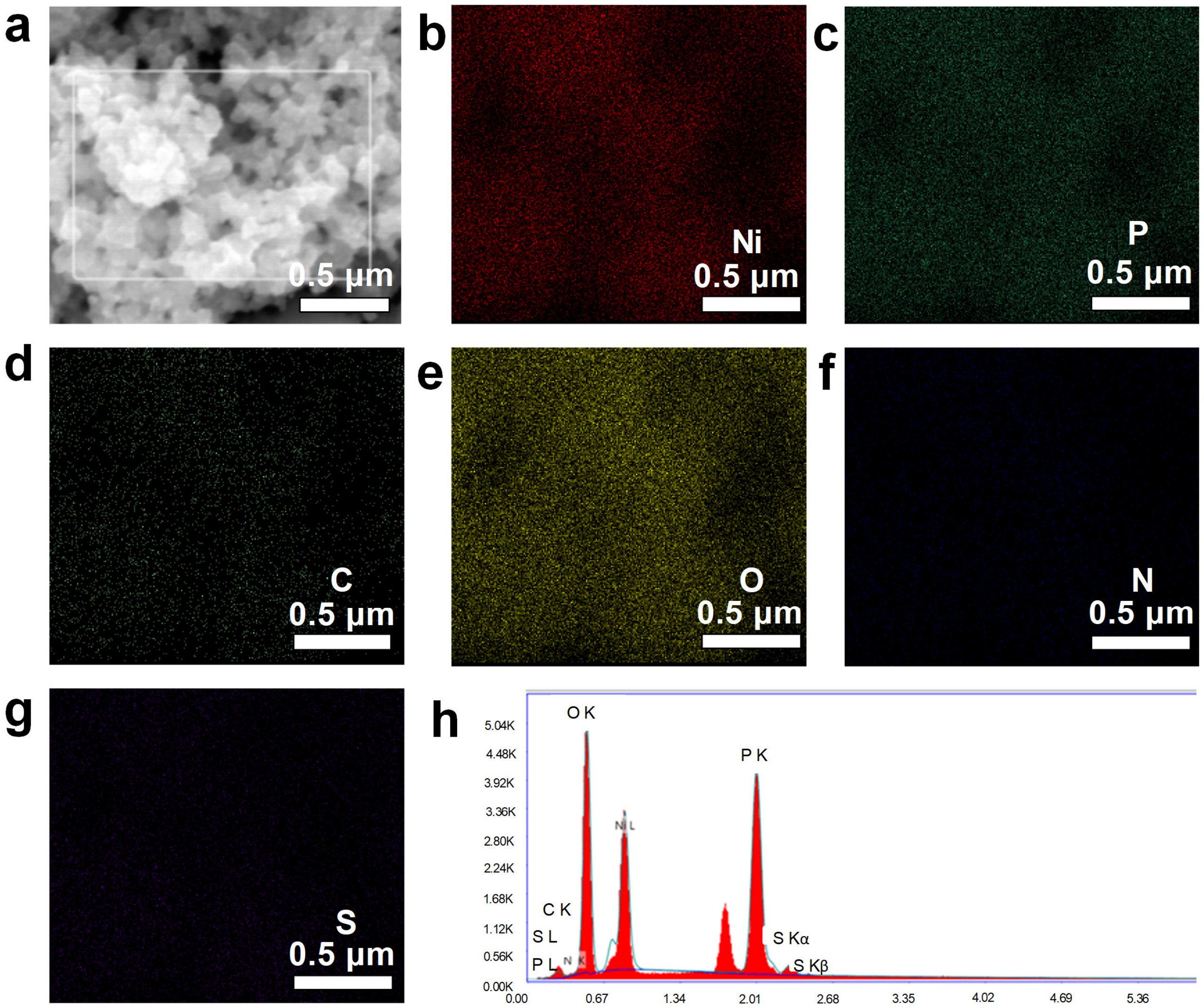

Supplementary Figure 7. The corresponding EDX elemental mapping images of Ni_2P/NiS@PCOS sample: (a)SEM images; (b) nickel (red); (c) phosphorus (green); (d) carbon (aqua); (e) oxygen (yellow); (f) nitrogen (blue); (g) sulfur (violet) in the selected area, respectively.

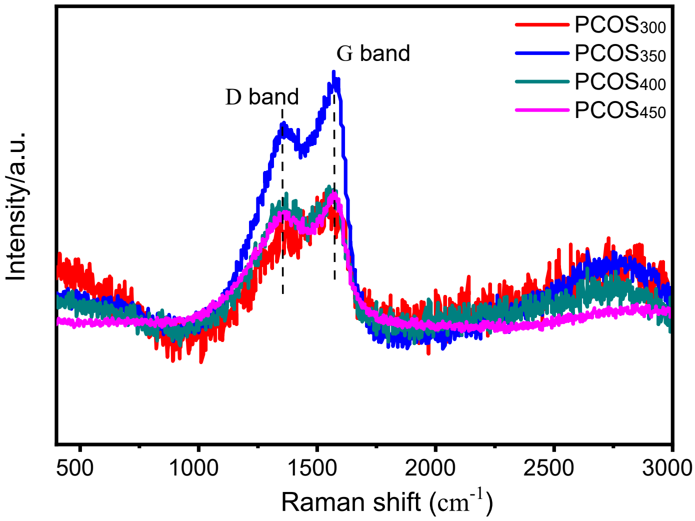

Supplementary Figure 8. Raman spectra of $PCOS_{300}$, $PCOS_{350}$, $PCOS_{400}$, $PCOS_{450}$, respectivety.

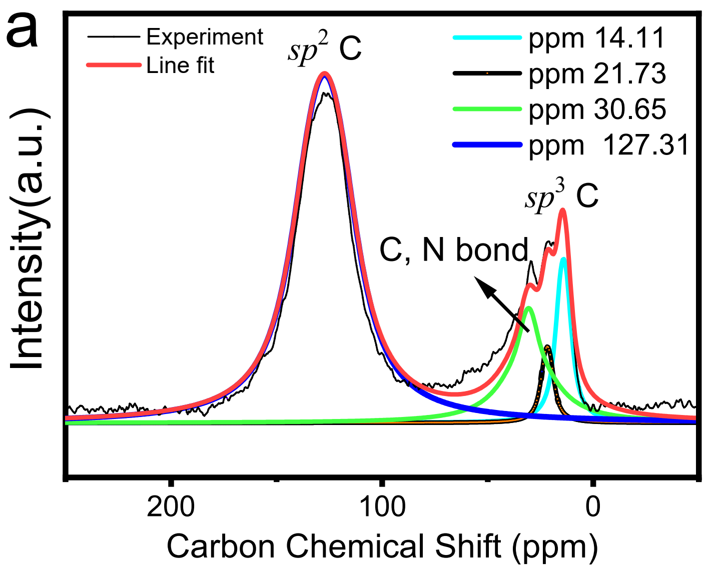

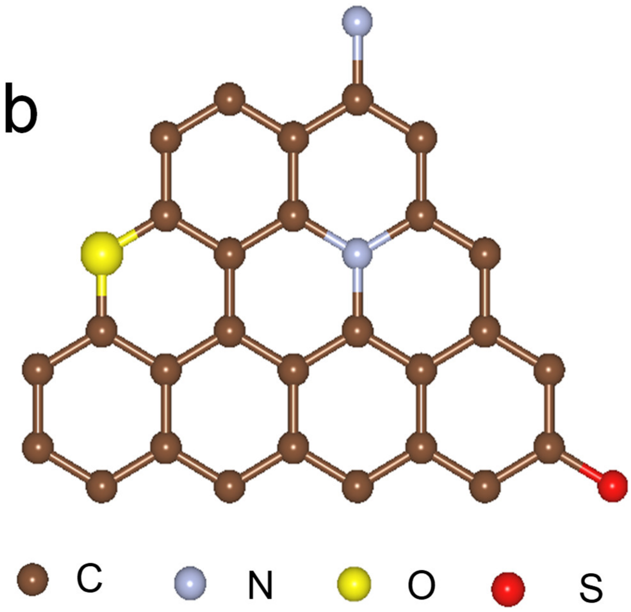

Supplementary Figure 9. (a) Solid-state ^{13}C-NMR of pure PCOS, (b) Schematic diagram of the molecular structure of PCOS.

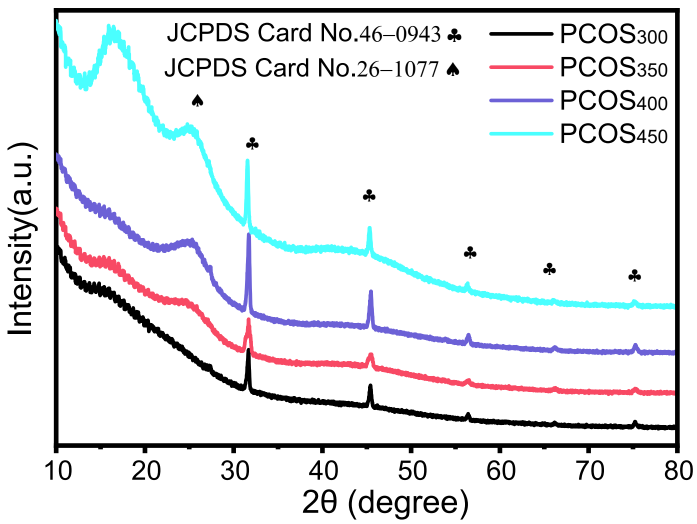

Supplementary Figure 10. XRD patterns of $PCOS_{300}$, $PCOS_{350}$, $PCOS_{400}$, $PCOS_{450}$, respectivety.

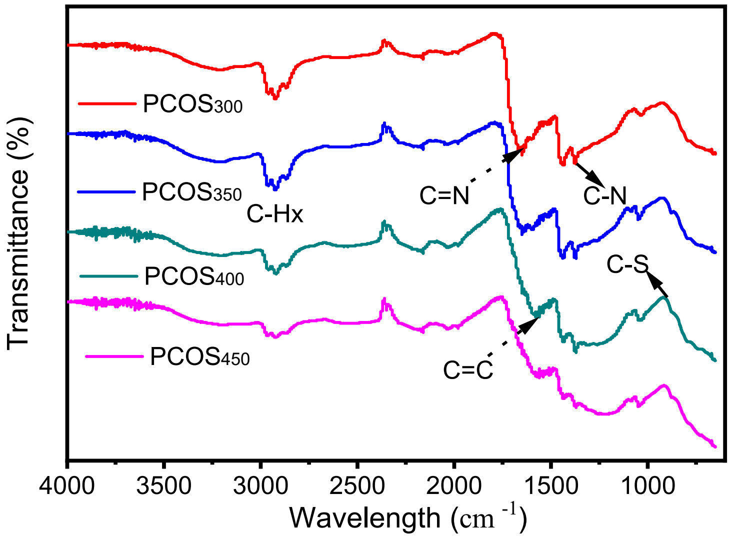

Supplementary Figure 11. FT-IR spectra of $PCOS_{300}$, $PCOS_{350}$, $PCOS_{400}$, $PCOS_{450}$, respectivety.

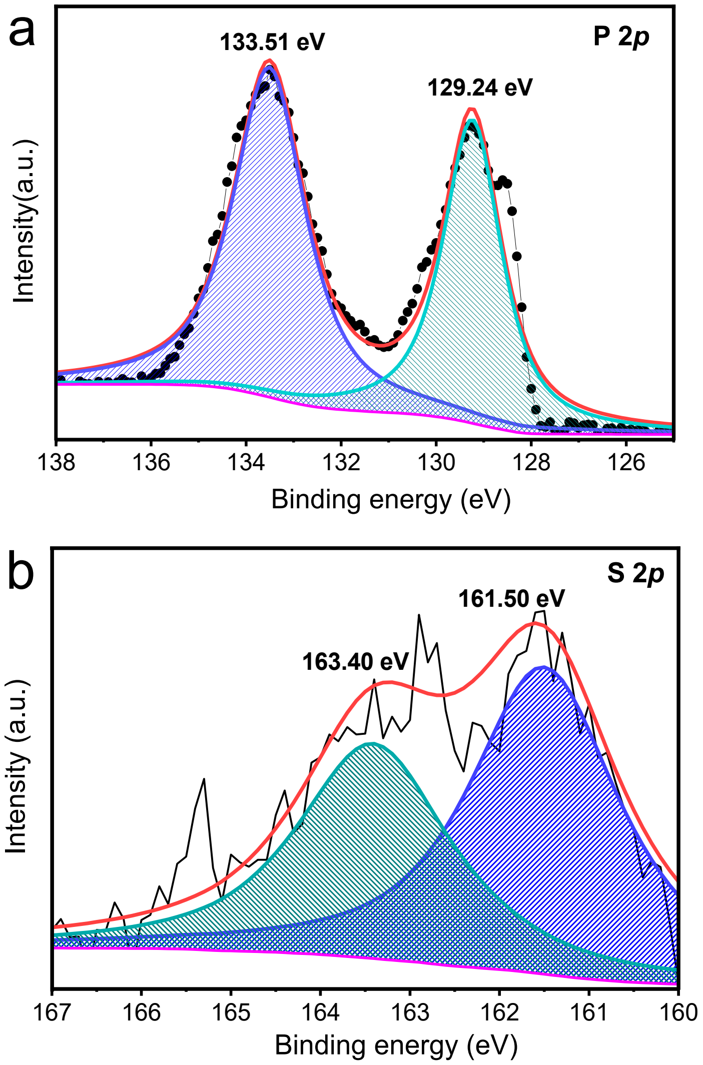

Supplementary Figure 12. XPS images of the Ni_2P/NiS@PCOS. (a) P 2p, (b) S 2p.

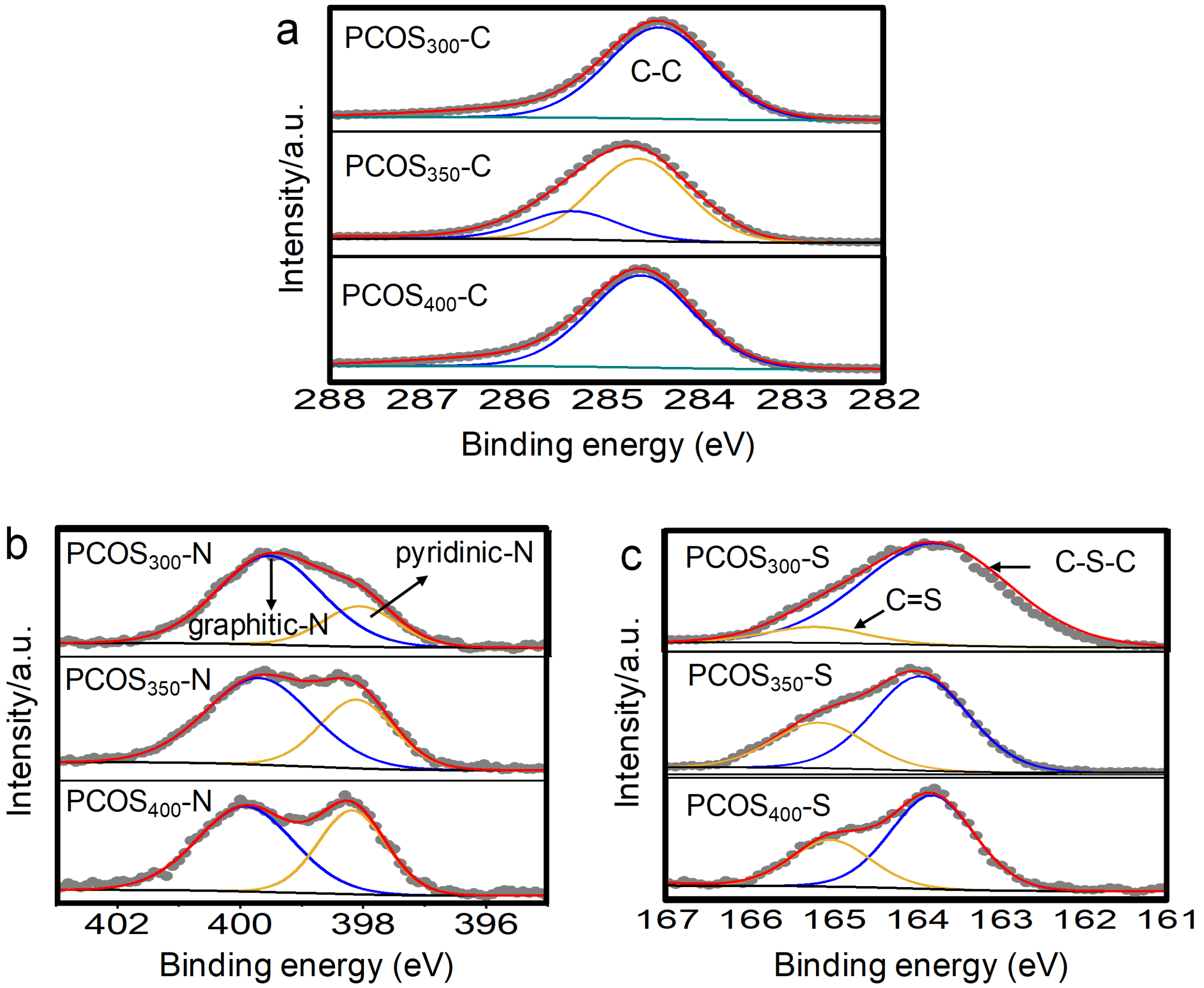

Supplementary Figure 13. XPS images of the polymeric carbon-oxygen semiconductor,which is the pure L-cysteine treated at 300 °C -400 °C for 3 h under nitrogen protection

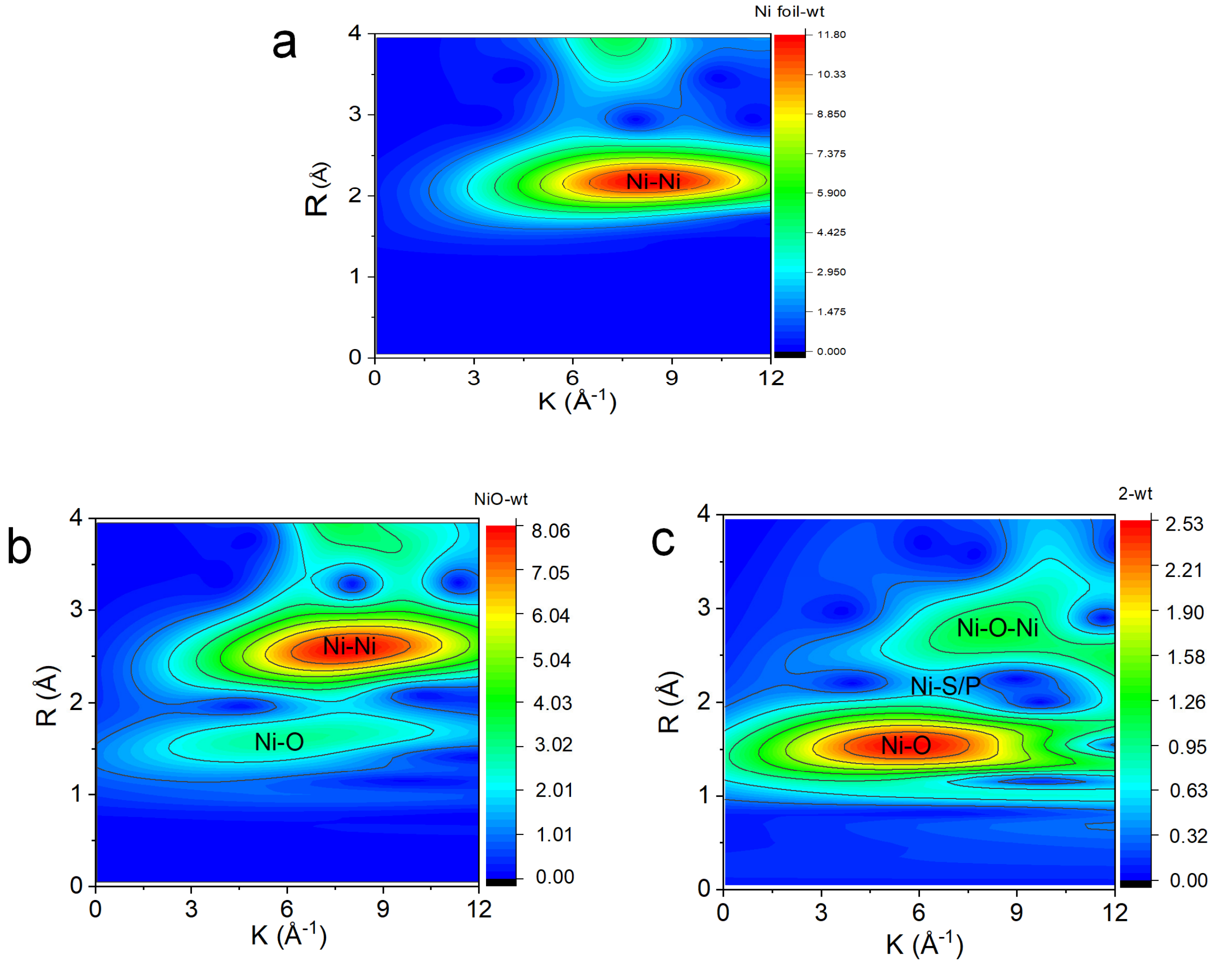

Supplementary Figure 14. Wavelet transform for the k2-weighted Ni K-edge EXAFS signal of (a) Ni standards, (b) NiO standards and (c) Ni_2P/NiS sample in this work.

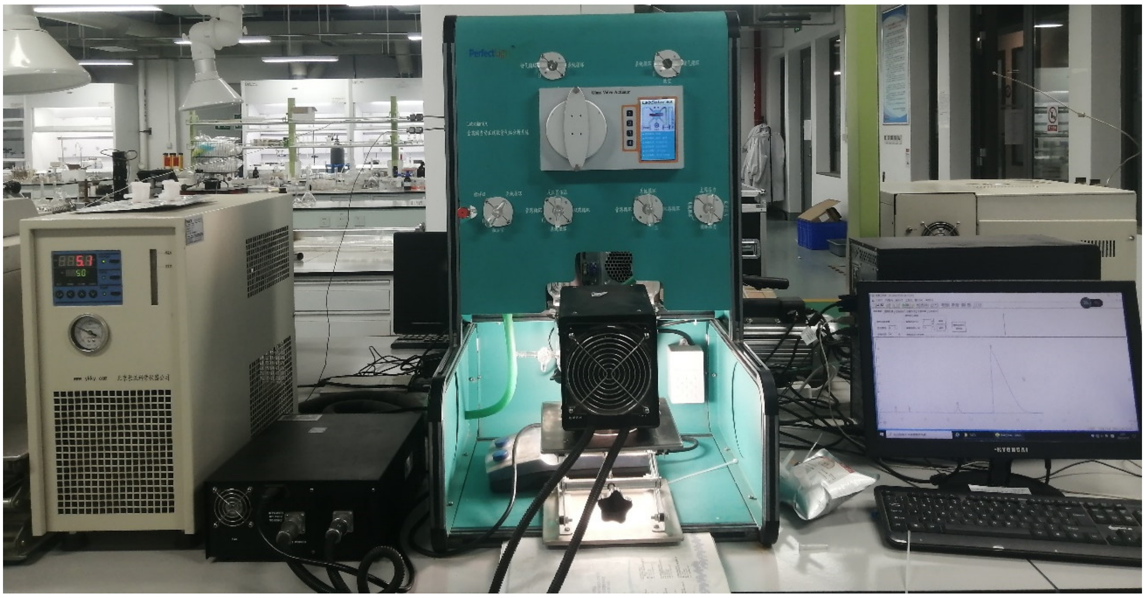

Supplementary Figure 15. Picture of photocatalytic overall water splitting test system.

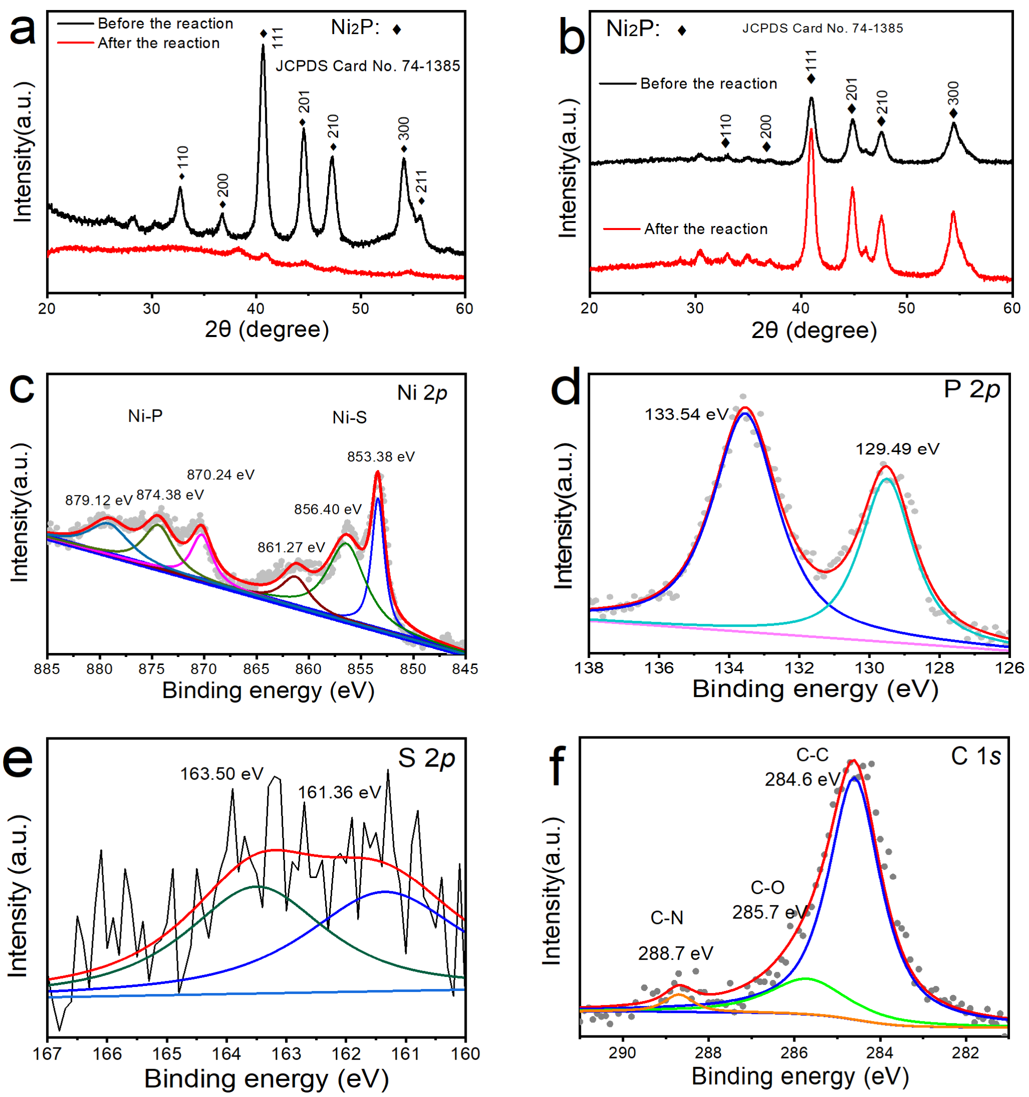

Supplementary Figure 16. (a) XRD patterns of the Ni_2P/NiS@PCOS samples without adding MnO_2 (before the reaction and after 3h reaction). (b) XRD patterns of the Ni_2P/NiS@PCOS samples with adding MnO_2 (before the reaction and after 3h reaction). (c-f) XPS spectra of the Ni_2P/NiS@PCOS samples with adding MnO_2 after 3h reaction.

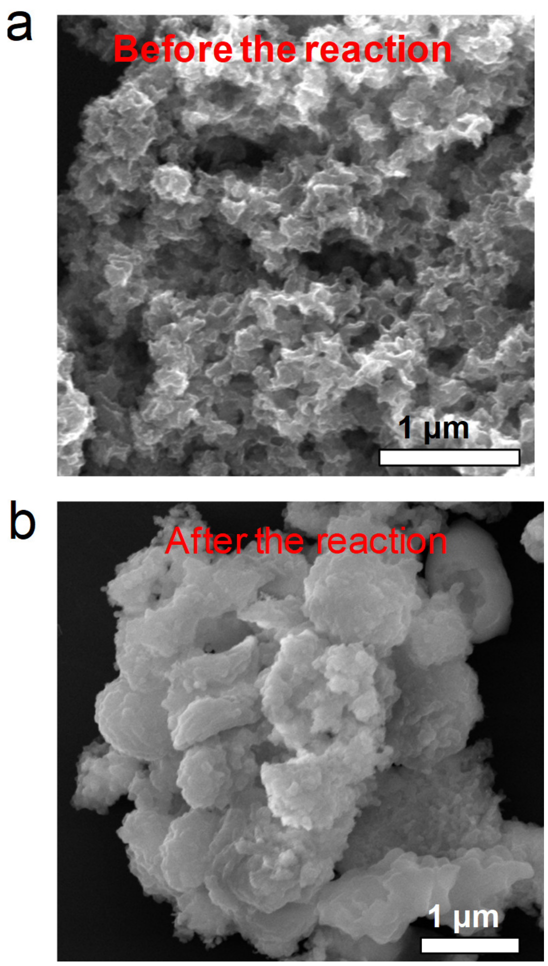

Supplementary Figure 17. The SEM of Ni_2P/NiS@PCOS sample without adding MnO_2, (a)before the reaction, (b)after 3h reaction.

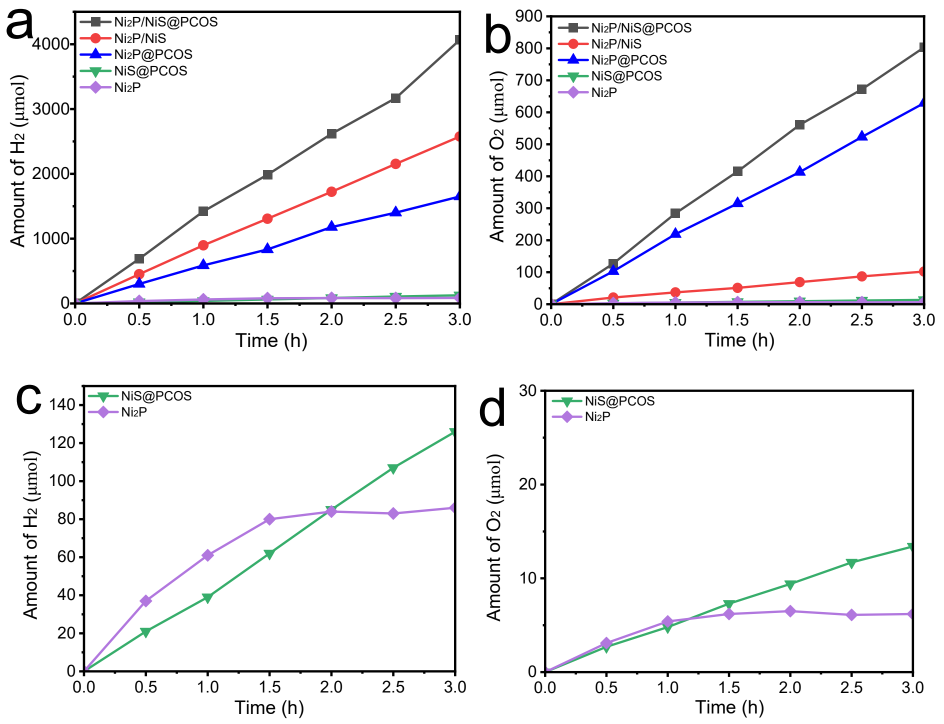

Supplementary Figure 18. (a,c) Time-dependent photocatalytic H_2 production of Ni_2P/NiS@PCOS, Ni_2P/NiS, Ni_2P/PCOS, NiS@PCOS and Ni_2P samples with lactic acid as holes sacrificial agents, (b,d) Time-dependent photocatalytic O_2 production of Ni_2P/NiS@PCOS, Ni_2P/NiS, Ni_2P/PCOS, NiS@PCOS and Ni_2P samples with $AgNO_3$ as electrons sacrificial agents. Experimental conditions: visible-light irradiation (λ>420 nm), 100 mg photocatalyst, 10 mg MnO_2, 100 mL deionized water.

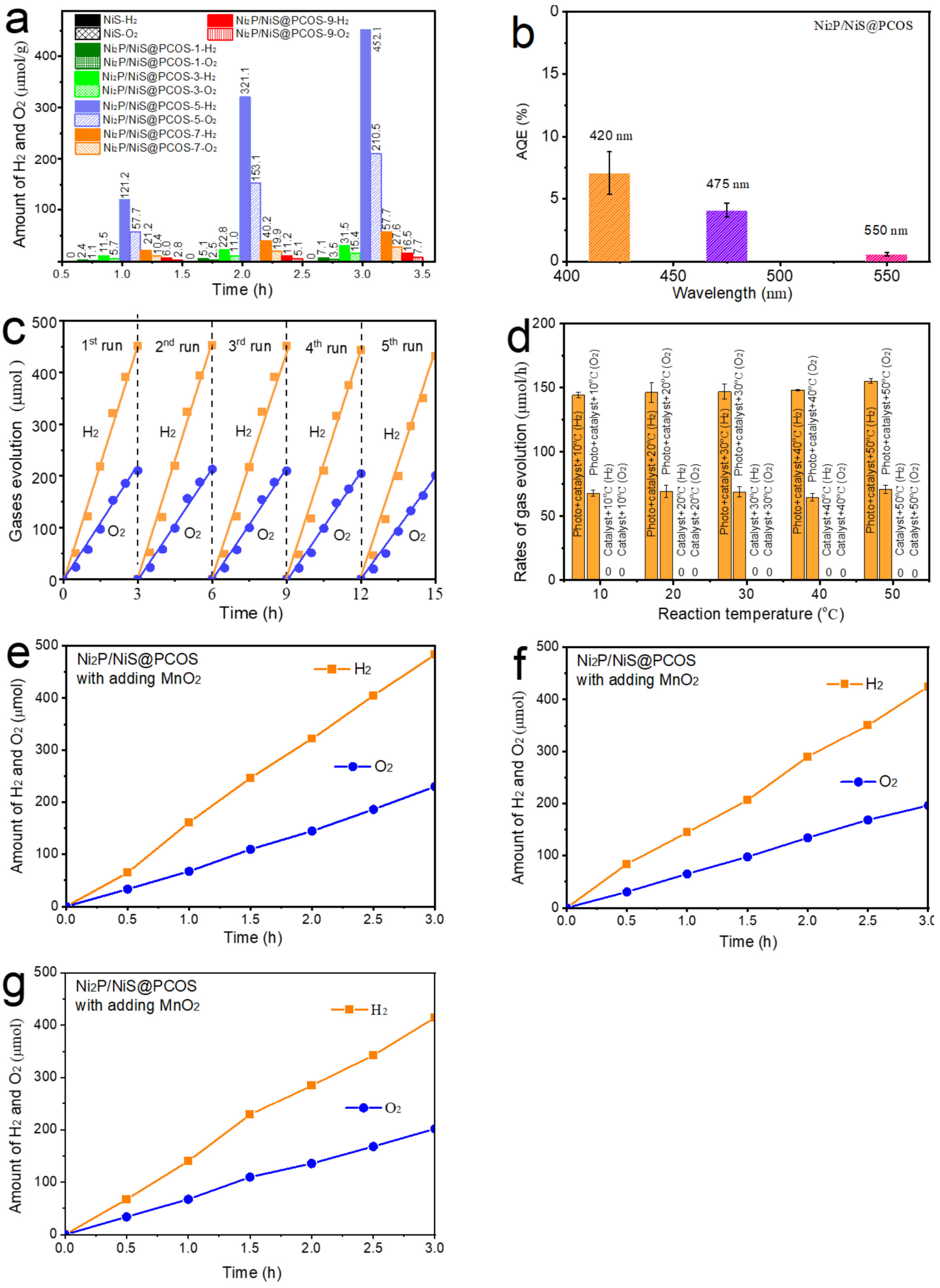

Supplementary Figure 19. (a) Time-dependent photocatalytic H_2 and O_2 production of $Ni_2P/NiS@PCOS$-x (0, 1, 3, 5, 7, 9) sample with MnO_2. (b) Wavelength-dependent AQE for photocatalytic H_2 evolution over $Ni_2P/NiS@PCOS$, (c)Stability tests of $Ni_2P/NiS@PCOS$ for 5 runs, (d) Hydrogen/oxygen production over $Ni_2P/NiS@PCOS$ under photothermocatalytic (10−50 °C) and thermo reaction (10−50 °C) conditions. (e-g) Three independent repeated experiments (tested by assistant professor He Li, PhD Baorong Xu and PhD Yuxi Ren. Experimental conditions: visible-light irradiation (λ>420 nm). photocatalyst 100 mg, deionized water 100 mL, and 10 mg MnO_2 assisted by continuous stirring.) Error bars indicate standard deviation for three measurements.

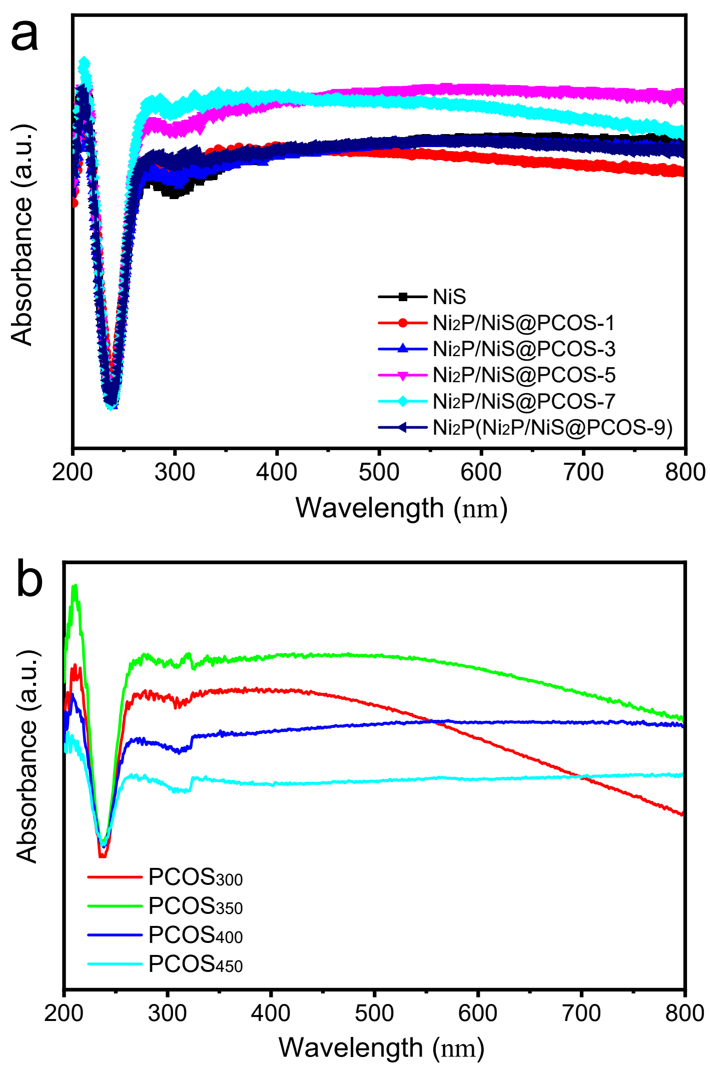

Supplementary Figure 20. UV–vis diffuse reflectance spectra (DRS) of Ni_2P, NiS, Ni_2P/NiS@PCOS and PCOS samples.

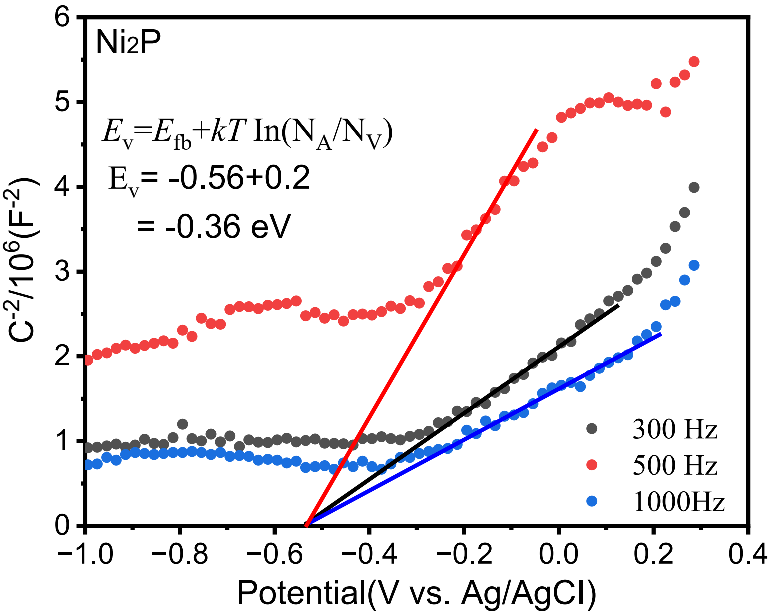

Supplementary Figure 21. Mott-Schottky plots of pure Ni_2P in 0.5 moL L^{-1} Na_2SO_4 electrolytes (pH=7).

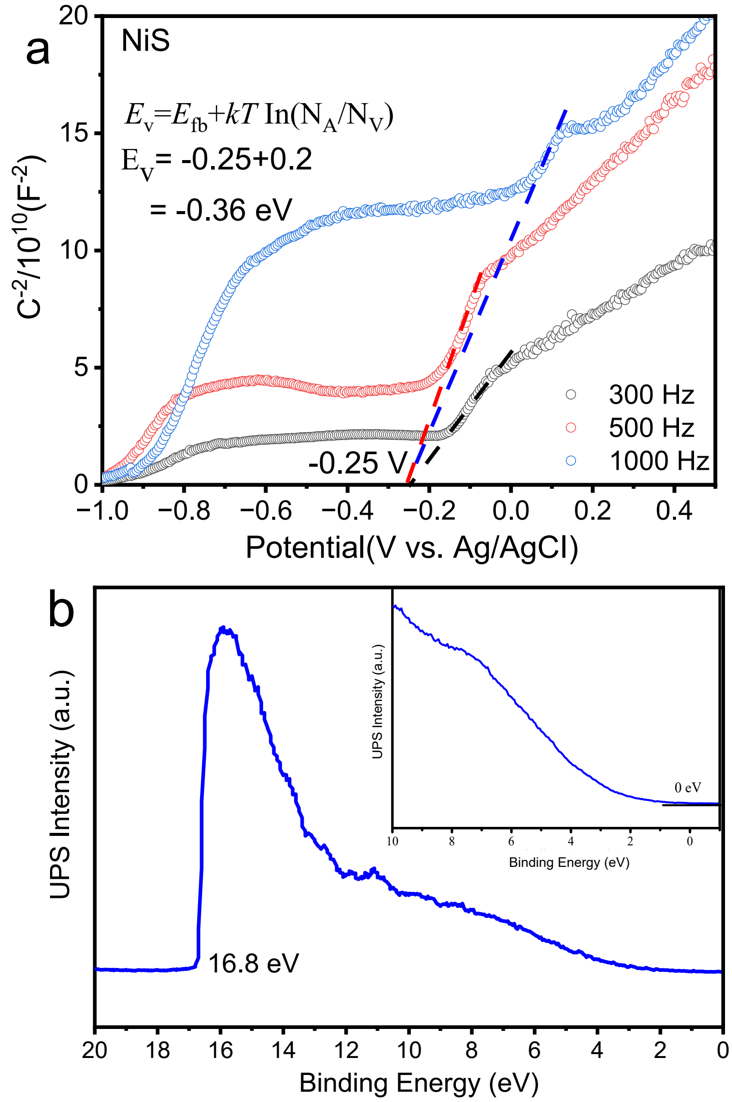

Supplementary Figure 22. Mott-Schottky plots of pure NiS in 0.5 moL L^{-1} Na_2SO_4 electrolytes (pH=7); (b)UPS spectra of pure NiS.

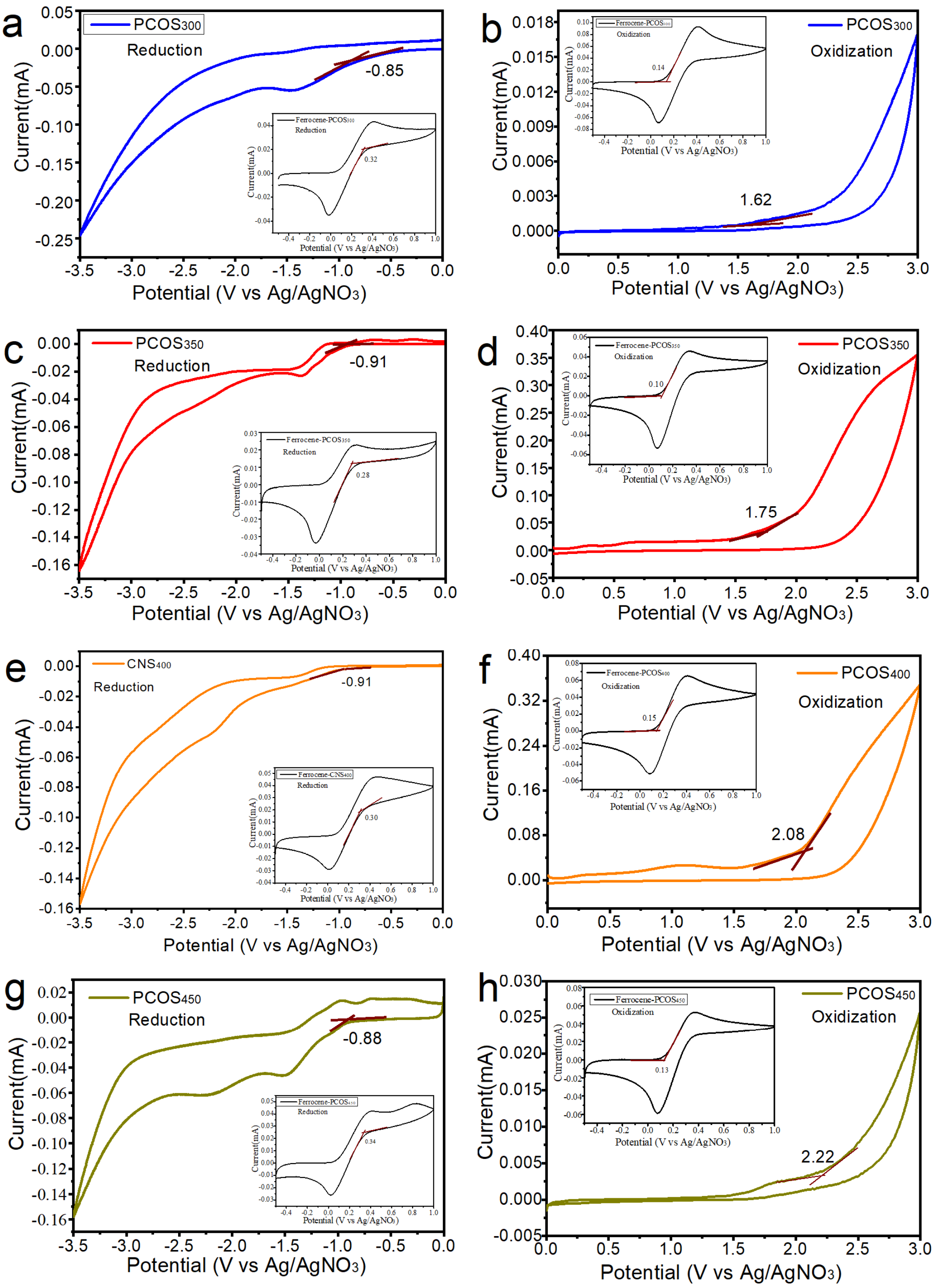

Supplementary Figure 23. Cyclic voltammograms of $PCOS_{300}$ (a-b), $PCOS_{350}$(c-d), $PCOS_{400}$(e-f), $PCOS_{450}$(g-h), respectivety.

a

$$E_{HOMO} = -(E^{ox}_{onset} - E_{ferrocene} + 4.8)\ eV$$

$$E_{LUMO} = -(E^{red}_{onset} - E_{ferrocene} + 4.8)\ eV$$

b

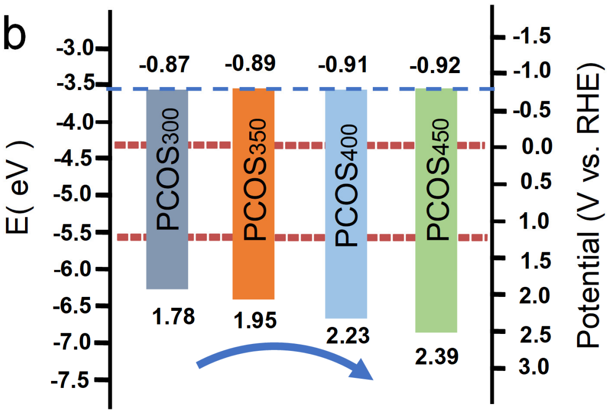

Supplementary Figure 24. (a-b) band structure diagram of $PCOS_{300}$, $PCOS_{350}$, $PCOS_{400}$, $PCOS_{450}$, respectivety.

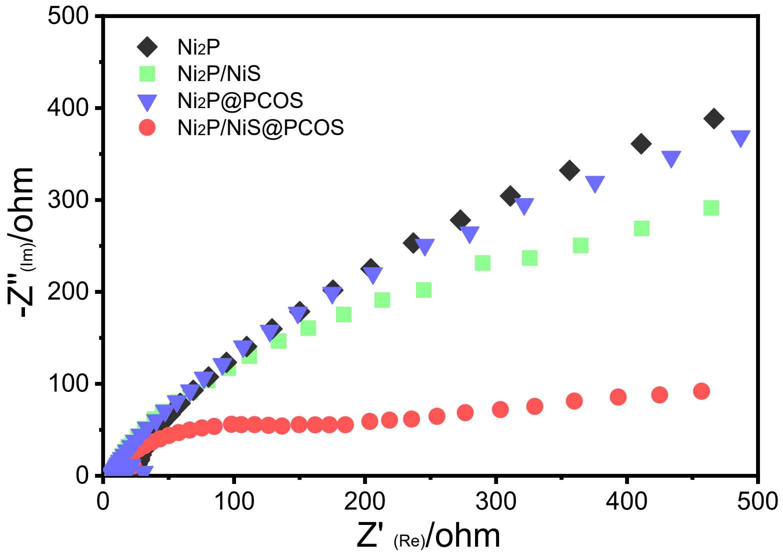

Supplementary Figure 25. Nyquist plots for the as-synthesized samples.

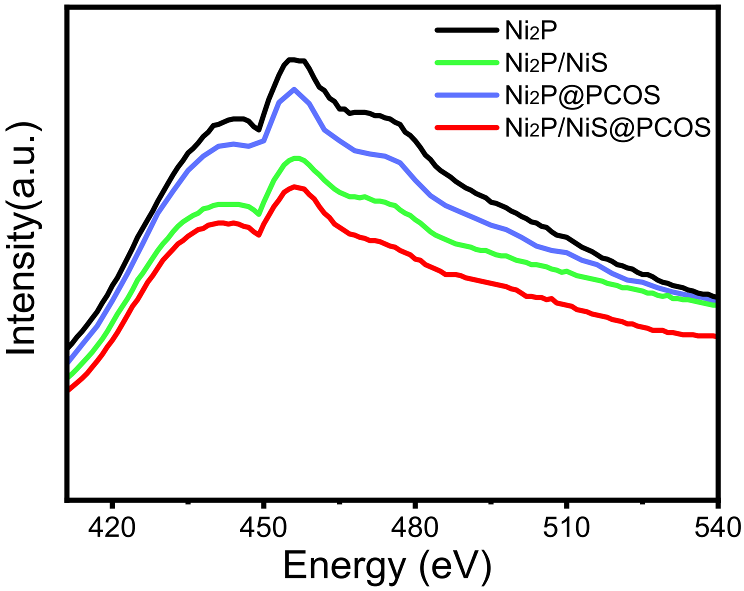

Supplementary Figure 26. PL spectra of various photocatalysts.

.

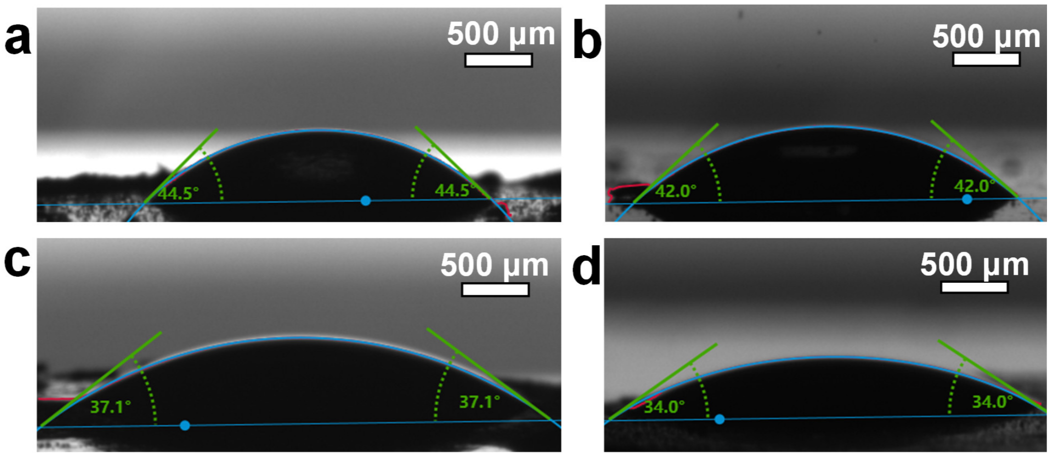

Supplementary Figure 27. Contact angle measurements of (a)Ni_2P, (b) Ni_2P/NiS, (c) Ni_2P@PCOS and (d) Ni_2P/NiS@PCOS.

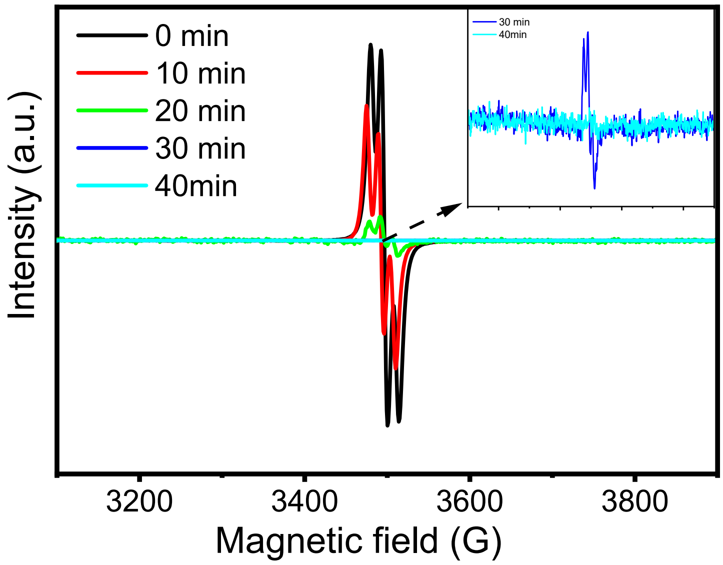

Supplementary Figure 28. ESR responses of the TEMPO-OH spin adduct in a Ni_2P water suspension under visible-light irradiation.

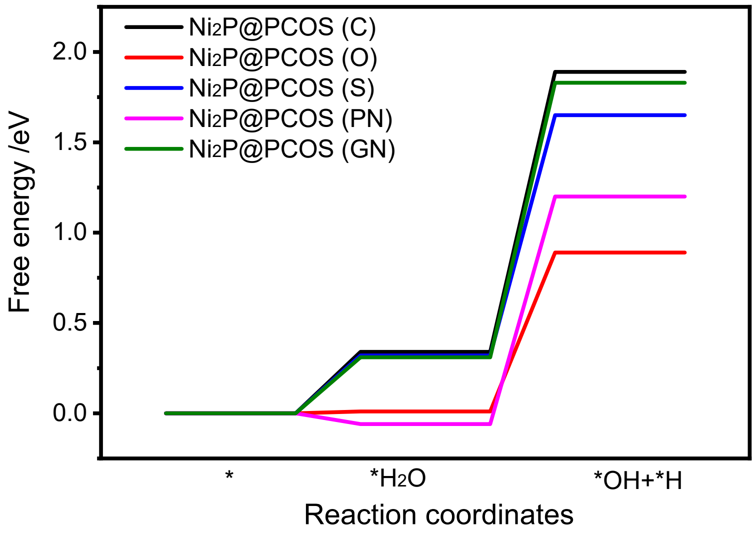

Supplementary Figure 29. Reaction energy for H_2O adsorption and dissociation on Ni_2P@PCOS structures.

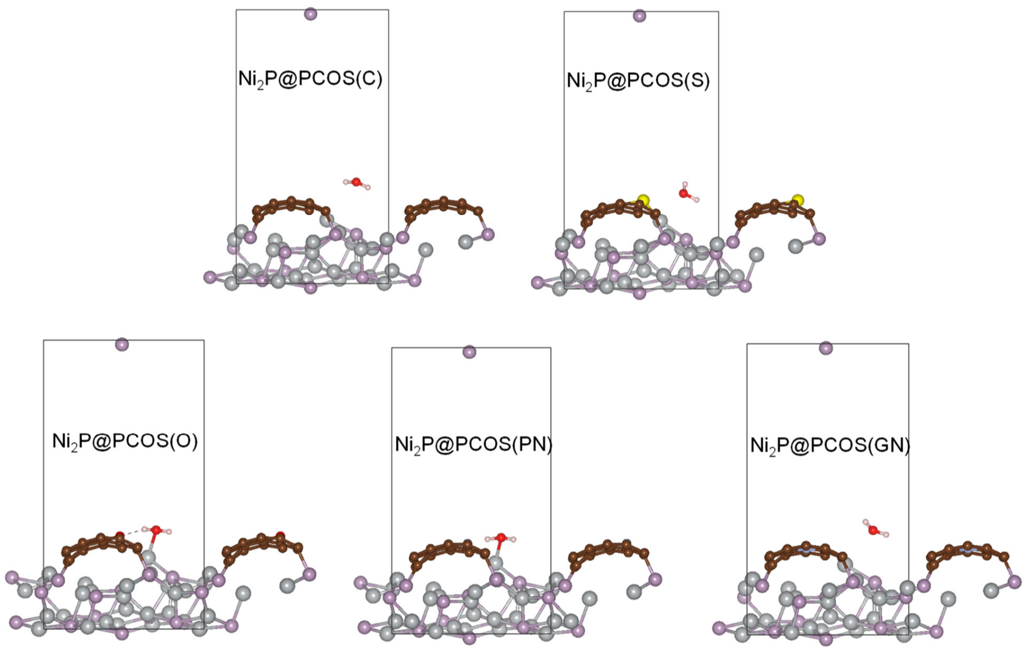

Supplementary Figure 30. Model diagram of adsorption water molecule on Ni_2P@PCOS surface. The yellow, red, pink, violet, brown and gray spheres represent S, O, H, P, C and Ni atoms, respectively.

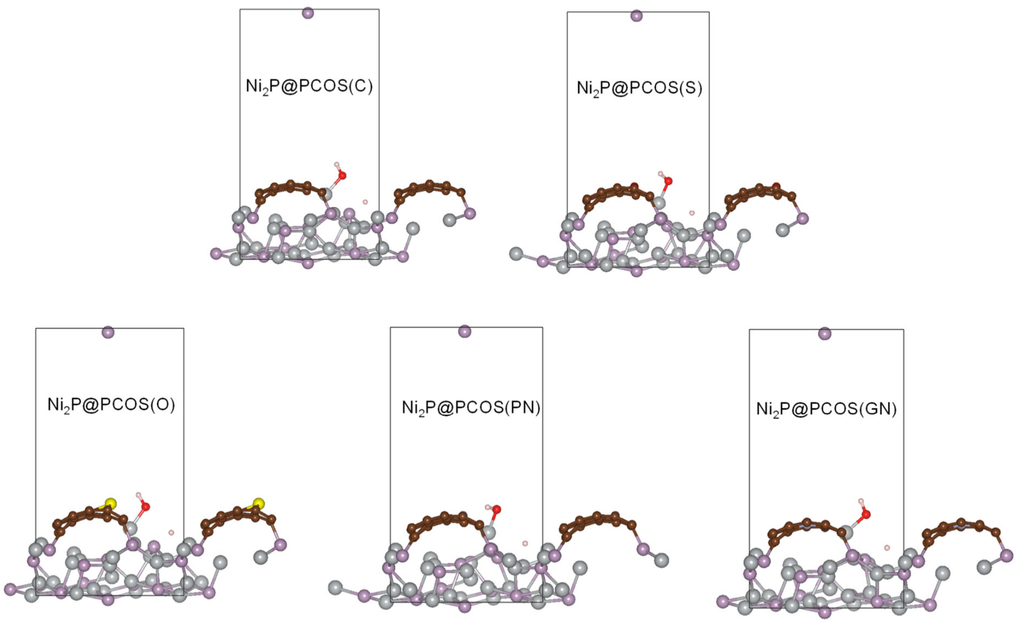

Supplementary Figure 31. Model diagram of dissociation water molecule on Ni_2P@PCOS surface. The yellow, red, pink, violet, brown and gray spheres represent S, O, H, P, C and Ni atoms, respectively.

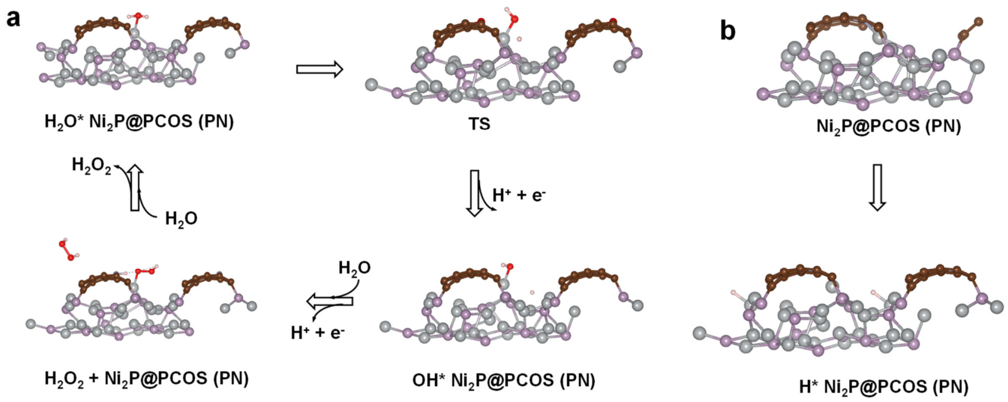

Supplementary Figure 32. (a) The optimized geometries of key reaction intermediates of 2e-mechanism of oxygen evolution reaction on Ni_2P@PCOS (pyridine nitrogen sites), (b) Hydrogen adsorption model diagram on Ni_2P@PCOS (pyridine nitrogen sites) surface. The yellow, red, pink, violet, brown and gray spheres represent S, O, H, P, C and Ni atoms, respectively.

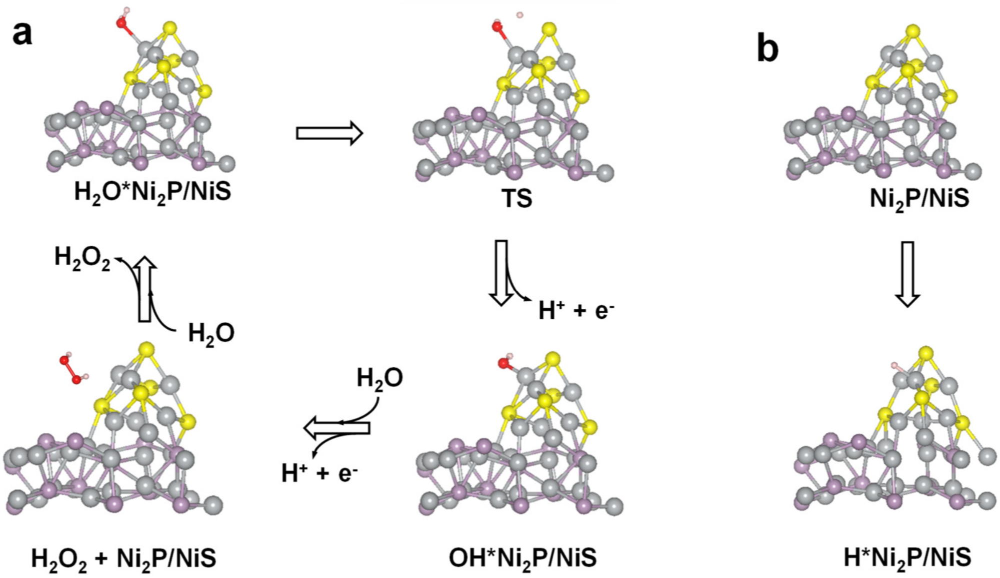

Supplementary Figure 33. (a) The optimized geometries of key reaction intermediates of 2e- mechanism of oxygen evolution reaction on Ni_2P/NiS, (b) Hydrogen adsorption model diagram on Ni_2P/NiS surface. The yellow, pink, violet and gray spheres represent S, H, P and Ni atoms, respectively.

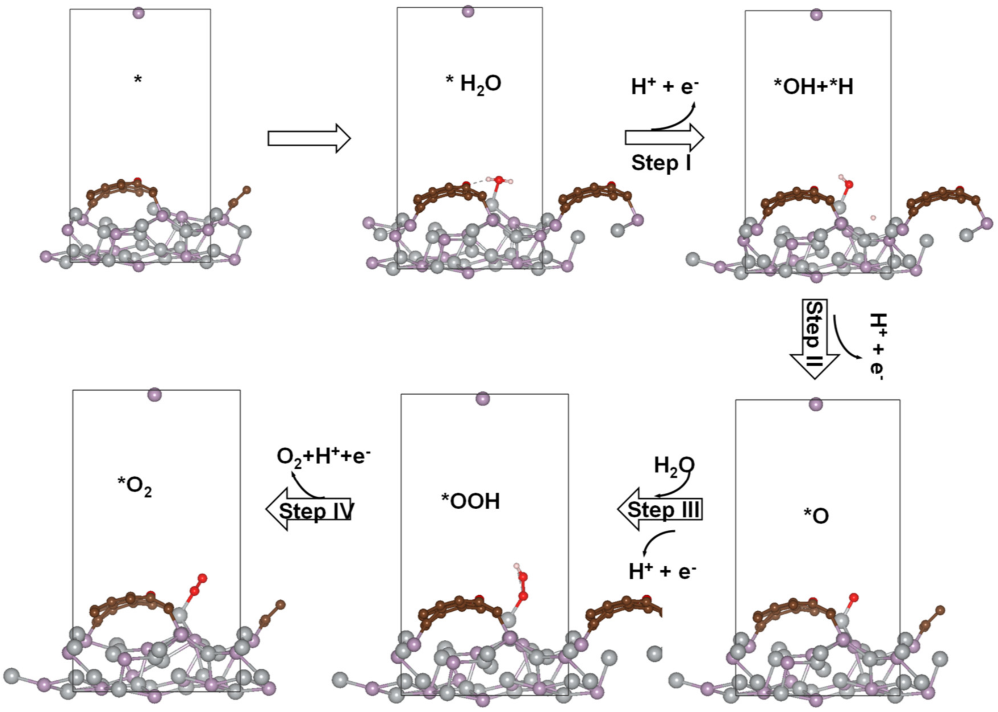

Supplementary Figure 34. The optimized geometries of key reaction intermediates of $4e^-$ mechanism of oxygen evolution reaction on Ni_2P@PCOS (O atom sites) surface. The yellow, red, pink, violet, brown and gray spheres represent S, O, H, P, C and Ni atoms, respectively.

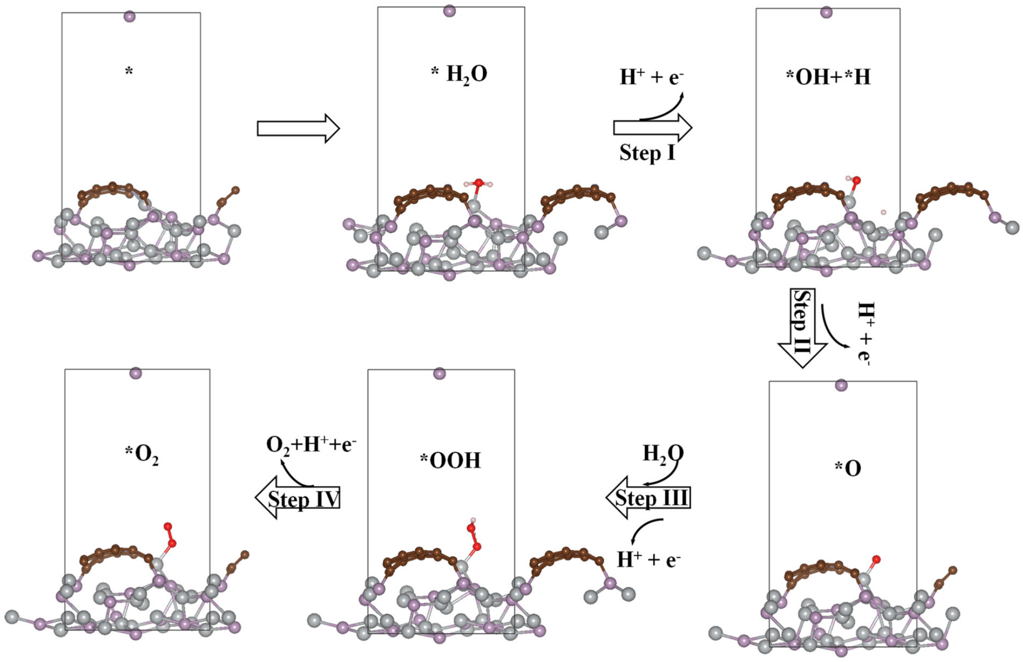

Supplementary Figure 35. The optimized geometries of key reaction intermediates of 4e$^-$ mechanism of oxygen evolution reaction on Ni_2P@PCOS (pyridine nitrogen sites) surface. The yellow, red, pink, violet, brown and gray spheres represent S, O, H, P, C and Ni atoms, respectively.

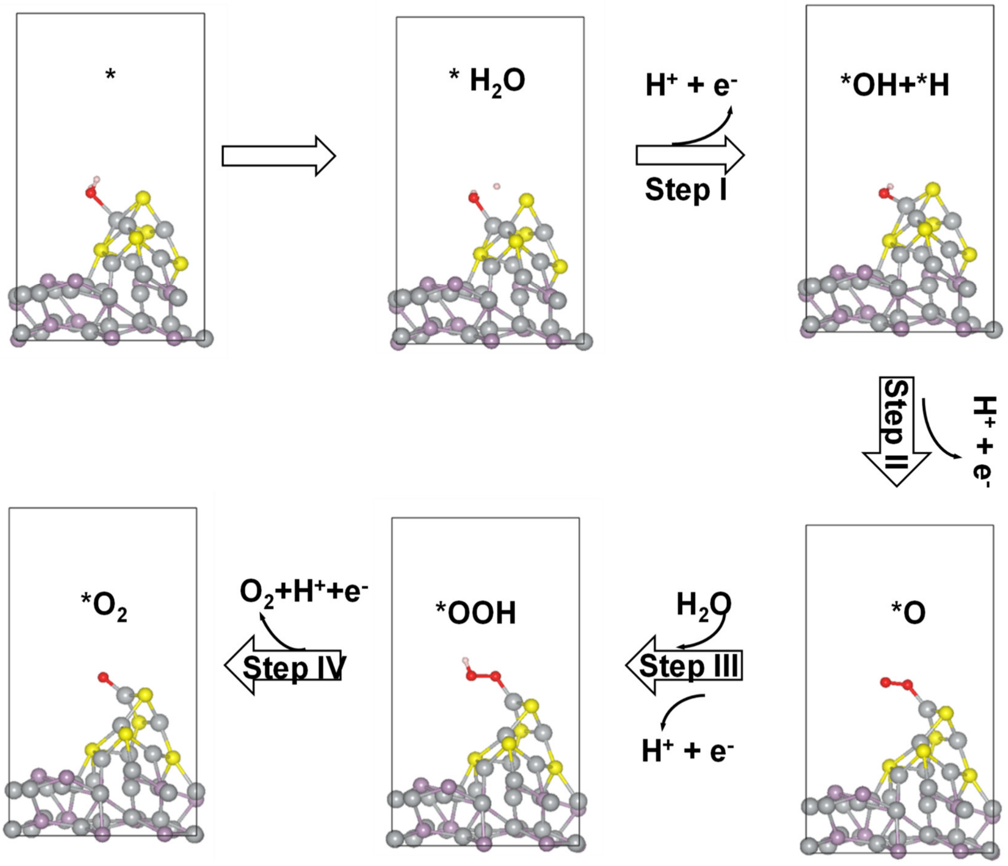

Supplementary Figure 36. The optimized geometries of key reaction intermediates of 4e$^-$ mechanism of oxygen evolution reaction on Ni_2P@NiS surface. The yellow, red, pink, violet and gray spheres represent S, O, H, P and Ni atoms, respectively.

Supplementary Table 1 BET surface area, pore volume and pore size of Ni_2P/NiS@PCOS sample.

Samples	BET surface area (m^2 g^{-1})	Pore volume (cm^3 g^{-1})	Pore width (nm)
Ni_2P/NiS@PCOS	29	0.2	1.33

Supplementary Table 2 The content of NiS, NiP and PCOS in the samples of Ni_2P/NiS@PCOS-x (0,1,3,5,7,9) were figured out by EDX.

samples	NiS	Ni_2P	PCOS
NiS@PCOS	61.66%	0%	38.34%
Ni_2P/NiS@PCOS-1	35.53%	29.53%	34.94%
Ni_2P/NiS@PCOS-3	23.50%	44.88%	31.62%
Ni_2P/NiS@PCOS-5	9.71%	52.88%	37.41%
Ni_2P/NiS@PCOS-7	2.66%	66.69%	30.65%
Ni_2P/NiS@PCOS-9	1.32%	71.06%	27.62%

Supplementary Table 3 Aomic percentage of polymeric carbon-oxygen semiconductor by XPS testing.

samples	C element	N element	S element	O element
$PCOS_{300}$	86.04 %	7.49 %	2.97 %	3.50 %
$PCOS_{350}$	82.39 %	6.44 %	2.87 %	8.30 %
$PCOS_{400}$	85.46 %	5.24 %	1.98 %	7.32 %

Supplementary Table 4 Aomic percentage of polymeric carbon-oxygen semiconductor by EDX testing.

samples	C element	N element	S element	O element
$PCOS_{300}$	67.7%	21 %	0.57%	10.6 %
$PCOS_{350}$	63.4 %	20.9 %	0.48 %	15.1 %
$PCOS_{400}$	67.4%	21 %	0.22 %	11.2 %

Supplementary Table 5. EXAFS fitting parameters at the Ni K-edge for various samples （S_0^2=0.75）

^aN: coordination numbers; ^bR: bond distance; $^c\sigma^2$: Debye-Waller factors; ^{d} ΔE_0: the inner potential correction. R factor: goodness of fit.

samples	shell	CN	R(Å)	σ^2	ΔE_0	R factor
Ni foil	Ni-Ni	12	2.48±0.01	0.0062	6.4±0.3	0.0012
Ni_2P/NiS/PCOS	Ni-P/S	4.7±0.2	2.23±0.01	0.0075	-5.1±1.3	0.0044
	Ni-Ni	2.9±0.2	2.61±0.02	0.0075		
	Ni-S-Ni	1.7±0.3	2.96±0.01	0.0075		
Ni_2P/NiS	Ni-O	5.1±0.4	2.01±0.01	0.0054	-7.7±1.9	0.0084
	Ni-S/P	0.7±0.2	2.33±0.03	0.0029		
	Ni-O-Ni	1.3±0.2	3.04±0.02	0.0026		

Supplementary Table 6 Comparison of the overall water splitting activity of $Ni_2P/NiS@PCOS$ and other typical materials.

Year	Catalyst	Light source	HER μmol $h^{-1}g^{-1}$	OER μmol $h^{-1}g^{-1}$	AQE	Author	Journal Title	Ref.
1980	$NiO\text{-}SrTiO_3$	_ 450 W	0.196	0.098	–	Domen K	Journal of the Chemical Society-Chemical Communications	1
1988	NiO(1wt%)-$K_4Nb_6O_{17}$	_ 450 W	66	38	–	Kudo A	Journal of catalysis	2
2001	Pt-loaded TiO_2-anatase/TiO_2-rutile	>300nm 400 W	180	90	–	Abe R	Chemical physics letters	3
2007	$(Ga_{1-x}Zn_x)(N_{1-x}O_x)RuO_2$	>400nm 450 W	200	100	30%, 600 nm	Maeda K	The Journal of Physical Chemistry C	4
2013	$IrO_2/Cr_2O_3/RuO_x/ZrO_2/TaON$	>400nm 450 W	14	7	0.1% 420nm	Maeda K	Chemistry–A European Journal	5
2015	CDots-C_3N_4	>420nm 300 W	8.4	4.1	16%, 420nm	Liu J	Science	6
2015	$In_{0.26}Ga_{0.74}N$: Mg	>400nm 300 W	1000	500	12.3% (400–475 nm) 200 °C	Kibria M G,	Nature communications	7
2016	Cr_2O_3/ $Ba_5Ta_4O_{15}$	UV 500 W	930	456	–	Julia Soldat	ChemCatChem	8
2016	Al-SrTiO3	> 300 nm	5500	2800	30%, 360nm	Ham Y	Journal of Materials Chemistry A	9
2017	PtOx/WN	>420nm 300 W	0.90	0.48	–	Yu Lei	Angewandte Chemie-International Edition	10
2018	aza-CMP/C_2N	420	5	2.5	4.3% 600 nm	Wang L	Angewandte Chemie-International Edition	11
2018	$Rh/Cr_2O_3\text{-}Ta_3N_5/KTaO_3$	>420nm 300 W	36.7	15.3	0.22% 420nm	Wang Z	Nature Catalysis	12
2018	RuDTC:Pt@CdS	460 ± 50 nm	170	71	0.27%	Wolff C M	Nature Energy	13
2019	Fe_2O_3/RGO/PCN	>420nm 300 W	1090	530	–	Pan Z	Angewandte Chemie-International Edition	14
2020	Pt@NH_2-UiO-66@MnOx	>400nm 300 W	19.6	10.1	–	Zhang J	Advanced Materials	15
2021	Pt/TiO_2/CdS-ZCGSe/Au/$BiVO_4$:Mo	>420nm 300 W	200	100	1.5% 420nm	Chen S,	Journal of the American Chemical Society	16
2021	BiOBr/C	>420nm 300 W	240	110	1.456% 420nm	Xiaoqi Zheng	ACS Nano	17
2021	$BDCNN_{350}/BDCNN_{425}$	>300nm 300 W	823.5	410.5	10.44% 420nm	Zhao D	Nature Energy	18

Supplementary Table 7. The calculated parameters about the corresponding energy-level structure of the different samples.

Samples	$E_{cut\ off}$/eV	W_s/eV	E /eV	E_{VB}/eV	E_g/eV	E_{CB}/eV
Ni_2P	16.70	-4.52	-1.90	-6.42	2.34	-4.08
NiS	16.80	-4.42	-0.00	-4.42	0.03	-4.39

Supplementary Table 8 Comparison of the photocatalytic hydrogen production activity of pure Ni_2P or CoxP in the half water splitting.

	Transition metal phosphide	Band gap	photocatalytic hydrogen production activity	References
1	Ni_2P	1.38 eV	untested	[19] Chemical Physics Letters 730 (2019) 478–484
2	Ni_2P	1.0 eV	16 μmol/10mg/h	[20] Catalysis Letters (2019) 149:3012–3026
3	Ni_2P	untested	12 mmol/g/h	[21] Chem.Commun., 2018, 54, 6188
4	Ni_2P	untested	2.19 μmol/10mg/h	[22] Journal of Colloid and Interface Science 537 (2019) 629–639
5	Ni_2P	untested	258.6 μmol/g/h	[23] *Chem. Eng. J.* 2021, 409, 128184
6	Ni_2P	untested	1250 μmol/g/h	[24] *Angew. Chem. Int. Ed.* 2018,57, 13082-13085
7	Co_2P	untested	136 μmol/8mg/h	[25] J. Phys. Chem. C 2016, 120, 6409−6415
8	Co_2P	0.74 eV	0.13 mmol/g/h	[26] ACS Appl. Mater. Interfaces 2019, 11, 22297-22306
9	CoP	1.71 eV	untested	[27] Adv. Energy Mater. 2017, 7, 1602355

Supplementary Table 9. The frequency of H_2O molecule at transfer states for dissociation on different sites.

Frequency	$f_{\mathrm{TS}_{\mathrm{Ni_2P@PCOS\,(O)}}}$/meV	$f_{\mathrm{TS}_{\mathrm{Ni_2P@PCOS\,(PN)}}}$/meV	$f_{\mathrm{TS}_{\mathrm{Ni_2P@NiS}}}$/meV
1 f	443.16	459.1634	466.1172
2 f	236.29	237.9481	118.3730
3 f	110.07	99.31787	84.3548
4 f	70.94	66.33454	72.2608
5 f	53.97	35.86591	58.0672
6 f	47.42	24.02567	48.0154
7 f	26.73	16.30477	15.0993
8 f	19.52	8.115175	3.4150
9 f/i	86.49	63.89422	179.3769

Supplementary Table 10. Adsorption energy of H_2O on Ni_2P@PCOS sites.

Site/doping element	Adsorption energy/eV
C	0.12
Ni	-0.08
Ni/O	-0.54
Ni/S	-0.11
Ni/pyridinic-N	-0.6
Ni/graphitic-N	-0.05

Supplementary Table 11. Gibbs free-energy value for the two steps of H_2O_2 evolution on Ni_2P@PCOS (PN)

Intermediate product	ΔE_{ads} /eV	ΔZPE /eV	TΔS /eV	ΔG /eV	G /eV
*	—	—	—	—	0
$*H_2O$	-0.619	0.045	-0.509	-0.065	-0.065
*TS	1.847	-0.158	0.063	1.626	1.560
*OH	1.377	-0.097	0.020	1.260	1.195
$*H_2O_2$	-1.004	0.213	0.082	-0.873	0.322
*	0.576	0.035	0.272	0.339	0.661

Supplementary Table 12. Gibbs free-energy value for the four steps of O_2 evolution on Ni_2P@PCOS (PN)

Intermediate product	ΔE_{ads} /eV	ΔZPE /eV	TΔS /eV	ΔG /eV	G /eV
*	—	—	—	—	0
$*H_2O$	-0.619	0.045	-0.509	-0.065	-0.065
*TS	1.847	-0.158	0.063	1.626	1.560
*OH	1.377	-0.097	0.020	1.260	1.195
*O	1.116	0.421	-0.006	1.543	2.738
*OOH	-1.628	0.374	0.114	-1.368	1.370
*OO	0.798	-0.299	-0.079	0.578	1.948
*	0.231	-0.023	0.497	-0.289	1.659

Supplementary Table 13. Gibbs free-energy value for the two steps of H_2O_2 evolution on Ni_2P@PCOS (O)

Intermediate product	ΔE_{ads} /eV	ΔZPE /eV	TΔS /eV	ΔG /eV	G /eV
*	—	—	—	—	0
*H_2O	-0.558	0.090	-0.478	0.010	0.010
*TS	1.355	-0.172	-0.027	1.210	1.220
*OH	1.07	-0.162	0.029	0.877	0.887
*H_2O_2	-0.407	0.193	0.071	-0.285	0.603
*	0.225	0.004	0.171	0.058	0.661

Supplementary Table 14. Gibbs free-energy value for the four steps of O_2 evolution on Ni_2P@PCOS (O)

Intermediate product	ΔE_{ads} /eV	ΔZPE /eV	TΔS /eV	ΔG /eV	G /eV
*	—	—	—	—	0
*H_2O	-0.558	0.090	-0.478	0.010	0.010
*TS	1.355	-0.172	-0.027	1.210	1.220
*OH	1.07	-0.162	0.029	0.877	0.887
*O	1.876	-0.460	-0.050	1.467	2.354
*OOH	-1.602	0.371	0.062	-1.293	1.061
*OO	0.982	-0.294	0.038	0.650	1.712
*	0.411	-0.026	0.438	-0.053	1.658

Supplementary Table 15. Gibbs free-energy value for the two steps of H_2O_2 evolution on Ni_2P/NiS

Intermediate product	ΔE_{ads} /eV	ΔZPE /eV	TΔS /eV	ΔG /eV	G /eV
*	—	—	—	—	0
*H_2O	-0.563	0.062	-0.454	-0.047	-0.047
*TS	2.117	-0.125	-0.068	2.060	2.013
*OH	-1.237	-0.190	0.076	-1.503	0.510
*H_2O_2	-0.667	0.104	-0.544	-0.019	0.491
*	0.680	0.037	0.547	0.170	0.661

Supplementary Table 16. Gibbs free-energy value for the four steps of O_2 evolution on Ni_2P/NiS

Intermediate product	ΔE_{ads} /eV	ΔZPE /eV	TΔS /eV	ΔG /eV	G /eV
*	—	—	—	—	0
*H_2O	-0.563	0.062	-0.454	-0.047	-0.047
*TS	2.117	-0.125	-0.068	2.060	2.013
*OH	-1.237	-0.190	0.076	-1.503	0.510
*O	1.469	-0.271	-0.069	1.267	1.777
*OOH	-1.901	0.125	-0.482	-1.294	0.483
*OO	0.919	-0.284	-0.058	0.693	1.176
*	1.037	-0.035	0.525	0.477	1.653

Supplementary Table 17. The atoms coordinate of Ni_2P/NiS model.

Atom	X coordinate/Å	Y coordinate/Å	Z coordinate/Å
Ni	2.6712	6.8513	0.7803
Ni	7.0032	1.7750	0.8200
Ni	0.2312	6.6445	3.1643
Ni	4.4987	1.6518	3.2679
Ni	2.5458	3.3068	1.0055
Ni	7.5734	8.4878	0.0830
Ni	8.8402	3.1403	3.2793
Ni	5.8460	8.3212	2.6307
Ni	3.8481	0.0321	0.0075
Ni	8.3138	5.1103	0.0898
Ni	1.1035	10.1393	2.0491
Ni	5.6371	4.9664	2.1959
Ni	0.0298	3.0862	0.8890
Ni	4.4979	8.2919	0.8668
Ni	1.8391	8.0759	2.6906
Ni	6.3548	2.8626	2.7609
Ni	0.2645	7.0228	0.8523
Ni	4.5219	1.9597	0.9476
Ni	1.7903	2.0398	2.7592
Ni	7.0583	6.8103	3.6700
Ni	3.9957	4.9780	0.7607
Ni	8.6021	0.1908	0.3688
Ni	1.4487	4.6020	2.6772
Ni	6.0548	0.3011	2.5205
Ni	2.0628	1.2585	0.4175
Ni	6.6343	6.6773	1.0319
Ni	4.0015	6.6434	2.5860
Ni	8.3165	1.1940	2.5944
Ni	2.0479	8.9315	0.3520
Ni	6.5144	3.9133	0.4513
P	3.7790	3.6813	2.9859
P	8.1384	8.6870	2.3571
P	1.5564	5.0596	0.1922
P	6.0673	10.1206	0.0577
P	3.4145	10.1338	2.4402
P	7.9436	4.9343	2.3949
P	4.9749	7.7461	4.5818
P	4.5076	5.9093	6.4366
P	2.8512	7.9325	6.1976
P	2.1678	5.4163	6.6963
P	5.3056	5.1230	4.2952
P	2.0248	6.2535	4.3675
S	0.8754	6.9918	6.1361
S	3.1577	8.8201	4.3008
S	3.2923	6.7232	7.9752
S	3.3246	4.5299	5.0460
S	6.4197	6.4315	5.6983

Supplementary Table 18. The atoms coordinate of Ni_2P@PCOS model.

Atom	X coordinate/Å	Y coordinate/Å	Z coordinate/Å
Ni	8.2803	4.9695	18.0444
Ni	3.7175	9.9689	0.0236
Ni	6.8432	1.7427	0.1983
Ni	7.4698	8.4305	0.4163
Ni	2.4084	6.7095	0.7554
Ni	3.1974	3.2740	0.8117
Ni	0.7048	3.3078	2.0688
Ni	1.1882	10.0117	2.3774
Ni	6.0022	4.9981	2.3931
Ni	5.9134	0.9607	3.1010
Ni	4.4953	8.0607	3.3194
Ni	0.5120	6.6761	3.3065
Ni	5.9245	9.8818	0.0121
Ni	8.7910	2.8059	0.1583
Ni	1.5432	4.6753	0.2441
Ni	0.1642	6.8878	0.2871
Ni	4.6418	1.7340	0.3437
Ni	6.4875	3.9696	0.4385
Ni	1.8544	8.9157	0.4561
Ni	6.5016	6.5301	0.8149
Ni	8.3698	0.2353	0.8549
Ni	2.1254	1.2208	0.8561
Ni	4.2731	8.0285	1.0459
Ni	4.1010	5.2498	1.1752
P	8.3312	5.0992	2.0732
P	7.4357	2.4903	2.2527
P	8.6775	8.3590	2.2874
P	5.0060	3.0463	2.3196
P	6.1673	9.1028	2.3695
P	2.0628	5.2040	2.7623
P	2.3294	7.8016	2.7501
P	2.6216	2.6054	2.8612
P	3.3644	-0.0543	2.9582
P	4.1400	6.0981	4.3004
P	6.7338	6.8678	3.4169
P	8.1525	0.3782	3.4867
C	3.4361	1.4612	4.3067
C	4.8579	1.6216	4.4362
C	0.1423	1.4243	4.8509
C	7.7740	1.4880	4.9678
C	2.5452	2.4662	4.9668
C	0.1264	6.0186	4.8904
C	7.6772	6.0610	4.9459
C	1.0603	2.4615	5.1665
C	5.5090	2.5904	5.2741

C	2.4879	4.9563	4.9962
C	6.9527	2.5606	5.4028
C	1.0411	4.9291	5.2512
C	3.2527	3.7388	5.2117
C	0.3052	3.6997	5.4886
C	6.9177	4.9676	5.4621
C	5.4626	4.9946	5.3614
C	4.7150	3.7779	5.4287
C	7.7427	3.7466	5.5983

Supplementary Table 19. Basic parameters of AQE test

wavelength (m)	activity (mol/h)	Avogadro number (mol^{-1})	light intensity (w/cm^2)	Area (cm^2)	Reaction Time (s)	Planck's constant (J s)	Speed of light (m/s)
λ	V	N_A	I	A	t	h	c
425*10^-9	34.7*10^-6	6.02*10^23	1.02^-3	78	3600	6.62*10^-34	3*10^8
475*10^-9	55.7*10^-6	6.02*10^23	2.38^-3	78	3600	6.62*10^-34	3*10^8
550*10^-9	7.1*10^-6	6.02*10^23	1.8410^-3	78	3600	6.62*10^-34	3*10^8

Supplementary Table 20. Basic parameters of STH test

Rate of hydrogen production	The Gibbs free energy	Optical power density	Area of illumination
R_{H2}	$\triangle G_r$	P_{sun}	S
3.84*10^-5 mmol s^{-1}	237000 J mol^{-1}	100 mW cm^{-2}	10 cm^2

Supplementary note 1: experimental details for reproducibility

(1) Preparation of the NiS@L-cysteine nanosheets: The NiS@L-cysteine nanosheets were prepared by a one-step hydrothermal process. In this procedure, 2 mmol $NiCl_2$ and 2 mmol L-Cysteine are mixed in 25 mL deionized water, and continuously stirring 6 h (450 r/min). Then, 10 mL solution with a certain concentration Na_2S was injected into above solution. And it should be emphasized that this process is critical. And the solution of Na_2S cannot be added quickly, in about 30 minutes. After continuously stirring 10 h, the mixed solution was then transferred into Teflon-lined stainless-steel autoclave and heated at 110°C for 2 h. After filtration and washing with deionized water several times, the resulting precipitant was dried using a vacuum freeze dryer overnight to finally obtain NiS@L-cysteine nanosheets.

(2) Preparation of Ni_2P/NiS@PCOS: 0.1 g NiS@L-cysteine nanosheets and sodium hypophosphite hydrate powder were respectively placed in the both end of a rectangle porcelain boat. And then the porcelain boat was tightly packaged with aluminum foil and put into a tube furnace. The porcelain boat must be tightly wrapped in aluminum foil, while ensuring that sodium hypophosphate and NaS do not mix. The furnace was charged with nitrogen as the carrying gas before being turned on, which was maintained at a flow rate of 20 mL/min, and the furnace reached the desired temperature (350°C) with a constant ramp rate of 5°C/min. After 180 min reaction, the Ni_2P/NiS@PCOS heterojunction was obtained.

Supplementary note 2: role of MnO_2

In the process of reaction, the crystal structure and microstructure of Ni_2P/NiS@PCOS were found to be severely etched after the reaction in the photocatalytic overall water splitting reaction when without any sacrificial agent or MnO_2. This is because H_2O_2 can etch the catalyst (ACS Catal. 2017, 7, 1637−1645). And the addition of MnO_2 to the reaction system is an effective way to quickly remove generated H_2O_2, thereby preventing photocatalyst poisoning (J. Propul. Power.1996, 12, 574-579). During this process, the formed H_2O_2 can be removed in time by adding MnO_2, which prevents the H_2O_2 from etching the catalyst. But once the etching has taken place, it cannot be recovered. So, the main function of MnO_2 is to decompose the H_2O_2 produced by photocatalysis into O_2, and the second function is to eliminate the formed H_2O_2 in a timely manner, thus preventing the etching of the catalyst by H_2O_2. So, when MnO_2 is added to the reaction system, Ni_2P/NiS@PCOS shows excellent and stable photocatalytic hydrogen and oxygen production rates (Figure 3a-b). We have also completed characterizing and analyzing the samples of Ni_2P/NiS@PCOS samples without and with adding MnO_2 after reaction by XRD tests. When adding MnO_2 in the system of reaction, the Ni_2P/NiS@PCOS sample exhibited better structural stability, which indicated that the H_2O_2 was decomposed in time by MnO_2, so the sample of Ni_2P/NiS@PCOS could not be etched (Supplementary Figure 16 a-f).

Supplementary notes 3: further discussion on the average lifetime

In the test of time-resolved fluorescence decay, the average lifetime (τ) of Ni_2P@PCOS is shorter than that of Ni_2P, but this result does not represent a contradiction with our design ideas. Due to the good charge separation effect, the fluorescence lifetime is long. But the fluorescence lifetime does not mean that the charge separation effect is ever excellent. The length of the electron lifetime is affected by various factors such as the charge separation efficiency, the electron mobility path length, and the interface reaction energy barrier. The reason why the average lifetime (τ) of Ni_2P@PCOS is shorter than that of Ni_2P may be attributed to the photostimulated electron migration path of Ni_2P@PCOS is shorter than that of Ni_2P. Thus, the average lifetime (τ) of Ni_2P@PCOS is shorter than Ni_2P. In order to adequately demonstrate that the charge separation effect of Ni_2P with NiS and PCOS, we further investigated the photocurrent test and the photoluminescence spectroscopy test. Figure 4b showed the Ni_2P/NiS@PCOS presented the strongest photocurrent response signals (about 155 μA cm^{-2}), which was about 8 and 31 times as high as that of the pure NiS (about 20.0 μA cm^{-2}) and Ni_2P@PCOS (about 5 μA cm^{-2}), respectively. Furthermore, the diameter of the Nyquist circle of Ni_2P/NiS@PCOS (Supplementary Figure 25) is much smaller than that of pure NiS and Ni_2P@PCOS. This proved that the Ni_2P/NiS@PCOS has ultrahigh electron conductivity and mobility. In addition, as shown in Supplementary Figure 26, the PL spectrum indicates that Ni_2P exhibits strong emission peaks, suggesting that electron-hole pairs recombine easily. But, in the PL spectrum of Ni_2P@PCOS, Ni_2P/NiS and Ni_2P/NiS@PCOS, a weaker emission peak at the same position can be observed, indicating that the addition of cocatalyst significantly inhibits the recombination of electron–hole pairs. In Figure 4d, the time-resolved fluorescence decay spectra also further illustrated the advantages of coating PCOS and NiS on the surface of Ni_2P for the improvement of charge separation. the average lifetime (τ) for Ni_2P/NiS@PCOS sample are increased, indicating that Ni_2P with double co-catalyst of NiS and PCOS possesses much longer lifetimes of photogenerated charge carriers than those of Ni_2P, Ni_2P@PCOS and Ni_2P/NiS.

REFERENCES

(1) Domen K, Naito S, Soma M, et al. Photocatalytic decomposition of water vapour on an NiO–$SrTiO_3$ catalyst[J]. *J. C. S. Chem. Comm.* **12**, 543-544 (1980).

(2) Kudo A, Tanaka A, Domen K, et al. Photocatalytic decomposition of water over NiO $K_4Nb_6O_{17}$ catalyst[J]. *J. Catal.* **111**, 67-76 (1988).

(3) Abe R, Sayama K, Domen K, et al. A new type of water splitting system composed of two different TiO_2 photocatalysts (anatase, rutile) and a IO_3^-/I^- shuttle redox mediator[J]. *Chem. Phys. Lett*. **344**, 339-344 (2001).

(4) Maeda K, Domen K. New non-oxide photocatalysts designed for overall water splitting under visible light[J]. *J. Phys. Chem. C.* **111**, 7851-7861 (2007).

(5) Maeda K, Lu D, Domen K. Direct water splitting into hydrogen and oxygen under visible light by using modified TaON photocatalysts with d0 electronic configuration[J]. *Chem. Eur. J.* **19**, 4986-4991 (2013).

(6) Liu J, Liu Y, Liu N, et al. Metal-free efficient photocatalyst for stable visible water splitting via a two-electron pathway[J]. *Science* **347**, 970-974 (2015).

(7) Kibria M G, Chowdhury F A, Zhao S, et al. Visible light-driven efficient overall water splitting using p-type metal-nitride nanowire arrays[J]. *Nat. Commun.* **6**, 1-8 (2015).

(8) Julia Soldat, G. Wilma Busser, Martin Muhler,and Michael Wark* Cr_2O_3 Nanoparticles on $Ba_5Ta_4O_{15}$ as a Noble-Metal-Free Oxygen Evolution Co-Catalyst for Photocatalytic Overall Water Splitting. *ChemCatChem*. **8**, 153–156 (2016).

(9) Ham Y, Hisatomi T, Goto Y, et al. Flux-mediated doping of $SrTiO_3$ photocatalysts for efficient overall water splitting[J]. *J. Mater. Chem. A.* **4**, 3027-3033 (2016).

(10) Yu Lei Wang, Ting Nie, Yu Hang Li, Xue Lu Wang, Li Rong Zheng, Ai Ping Chen, Xue Qing Gong, and Hua Gui Yang*.Black Tungsten Nitride as aMetallic Photocatalystfor Overall WaterSplitting Operable at up to 765 nm. *Angew. Chem. Int. Ed.* **56**, 7430–7434 (2017).

(11) Wang L, Zheng X, Chen L, et al. Van der Waals Heterostructures Comprised of Ultrathin Polymer Nanosheets for Efficient Z-Scheme Overall Water Splitting[J]. *Angew. Chem. Int. Ed.* **130**, 3512-3516 (2018).

(12) ZhengWang, Kazunari Domen et al. Overall water splitting by Ta_3N_5 nanorod single crystals grown on the edges of $KTaO_3$ particles. *Nat. Catal. 1*, 756-763 (2018).

(13) Wolff C M, Frischmann P D, Schulze M, et al. All-in-one visible-light-driven water splitting by combining nanoparticulate and molecular co-catalysts on CdS nanorods[J]. *Nat. Energy*. **3**, 862-869 (2018).

(14) Pan Z, Zhang G, Wang X. Polymeric carbon nitride/reduced graphene oxide/Fe_2O_3: all-solid-state Z-scheme system for photocatalytic overall water splitting[J]. *Angew. Chem. Int. Ed.* **131**, 7176-7180 (2019).

(15) Zhang J, Bai T, Huang H, et al. Metal–Organic-Framework-Based Photocatalysts Optimized by Spatially Separated Cocatalysts for Overall Water Splitting[J]. *Adv. Mater.* **32**, 2004747 (2020).

(16) Chen S, Vequizo J J M, Pan Z, et al. Surface Modifications of $(ZnSe)_{0.5}$ $(CuGa_{2.5}Se_{4.25})_{0.5}$ to Promote Photocatalytic Z-Scheme Overall Water Splitting[J]. *J. Am. Chem. Soc.* **143**, 10633-10641 (2021).

(17) Xiaoqi Zheng, et al, High Carrier Separation Efficiency in Morphology-Controlled BiOBr/C Schottky Junctions for Photocatalytic Overall Water Splitting, *ACS Nano*. **15**, 13209-13219 (2021).
(18) Zhao D, Wang Y, Dong C L, et al. Boron-doped nitrogen-deficient carbon nitride-based Z-scheme heterostructures for photocatalytic overall water splitting[J]. *Nat. Energy*, **6**, 388-397 (2021).
(19) Farahi E, Memarian N. Nanostructured nickel phosphide as an efficient photocatalyst: effect of phase on physical properties and dye degradation[J]. *Chem. Phys. Lett.,* **730**: 478-484 (2019).
(20) Zhao S, Xu J, Yu H, et al. RGO boosts band gap regulates for constructing Ni_2P/RGO/MoO_2 Z-scheme heterojunction to achieve high efficiency photocatalytic H_2 evolution[J]. *Catal. Lett.*, **149**, 3012-3026 (2019).
(21) Lou Y, He J, Liu G, et al. Efficient hydrogen evolution from the hydrolysis of ammonia borane using bilateral-like WO_{3-x} nanorods coupled with Ni_2P nanoparticles[J]. *Chem. Commun.*, **54**, 6188-6191 (2018).
(22) Li Y, Jin Z, Wang H, et al. Effect of electron-hole separation in MoO_3@Ni_2P hybrid nanocomposite as highly efficient metal-free photocatalyst for H_2 production[J]. *J. Colloid. Interf. Sci.*, **537**, 629-639 (2019).
(23) Liu Y, Zhao Y, Wu Q, et al. Charge storage of carbon dot enhances photo-production of H_2 and H_2O_2 over Ni_2P/carbon dot catalyst under normal pressure[J]. *Chem. Eng. J.*, **409**, 128184 (2021).
(24) Li S H, Zhang N, Xie X, et al. Stress-Transfer-Induced In Situ Formation of Ultrathin Nickel Phosphide Nanosheets for Efficient Hydrogen Evolution[J]. *Angew. Chem. Int. Edit.*, **130**, 13266-13269 (2018).
(25) Tian B, Li Z, Zhen W, et al. Uniformly sized (112) facet Co_2P on graphene for highly effective photocatalytic hydrogen evolution[J]. *J. Phys. Chem. C.*, **120**, 6409-6415 (2016).
(26) Li N, Ding Y, Wu J, et al. Efficient, full spectrum-driven H_2 evolution Z-scheme Co_2P/CdS photocatalysts with Co–S bonds[J]. *ACS Appl. Mater. Inter.*, **11**, 22297-22306 (2019).
(27) Xue Z H, Su H, Yu Q Y, et al. Janus Co/CoP nanoparticles as efficient Mott–Schottky electrocatalysts for overall water splitting in wide pH range[J]. *Adv. Energy. Mater.*, **7**, 1602355 (2017).
